# Identification of Methylated Genes Associated with Aggressive Bladder Cancer

**DOI:** 10.1371/journal.pone.0012334

**Published:** 2010-08-23

**Authors:** Carmen J. Marsit, E. Andres Houseman, Brock C. Christensen, Luc Gagne, Margaret R. Wrensch, Heather H. Nelson, Joseph Wiemels, Shichun Zheng, John K. Wiencke, Angeline S. Andrew, Alan R. Schned, Margaret R. Karagas, Karl T. Kelsey

**Affiliations:** 1 Department of Pathology and Laboratory Medicine, Brown University, Providence, Rhode Island, United States of America; 2 Department of Community Health, Center for Environmental Health and Technology, Brown University, Providence, Rhode Island, United States of America; 3 Department of Neurological Surgery, University of California San Francisco, San Francisco, California, United States of America; 4 Division of Epidemiology and Community Health, Masonic Cancer Center, University of Minnesota, Minneapolis, Minnesota, United States of America; 5 Section of Biostatistics and Epidemiology, Department of Community and Family Medicine, Dartmouth Medical School, Lebanon, New Hampshire, United States of America; 6 Department of Pathology, Dartmouth Medical School, Lebanon, New Hampshire, United States of America; Oregon State University, United States of America

## Abstract

Approximately 500,000 individuals diagnosed with bladder cancer in the U.S. require routine cystoscopic follow-up to monitor for disease recurrences or progression, resulting in over $2 billion in annual expenditures. Identification of new diagnostic and monitoring strategies are clearly needed, and markers related to DNA methylation alterations hold great promise due to their stability, objective measurement, and known associations with the disease and with its clinical features. To identify novel epigenetic markers of aggressive bladder cancer, we utilized a high-throughput DNA methylation bead-array in two distinct population-based series of incident bladder cancer (n = 73 and n = 264, respectively). We then validated the association between methylation of these candidate loci with tumor grade in a third population (n = 245) through bisulfite pyrosequencing of candidate loci. Array based analyses identified 5 loci for further confirmation with bisulfite pyrosequencing. We identified and confirmed that increased promoter methylation of *HOXB2* is significantly and independently associated with invasive bladder cancer and methylation of *HOXB2*, *KRT13* and *FRZB* together significantly predict high-grade non-invasive disease. Methylation of these genes may be useful as clinical markers of the disease and may point to genes and pathways worthy of additional examination as novel targets for therapeutic treatment.

## Introduction

In the United States in 2009, an estimated 71,000 cancers of the urinary bladder were diagnosed and greater than 14,000 deaths were attributed to this disease [1]. The vast majority of deaths occur in patients with incident high stage, high grade, invasive tumors that infiltrate the muscular layers of the bladder. Low grade, non-invasive disease, on the other hand, can be successfully treated, though this success comes at great economic burden to the healthcare system. Approximately 500,000 patients require monitoring in the U.S. leading to estimated diagnosis to death per patient costs ranging from $96,000 to $187,000, thereby resulting in $2.2 billion in annual expenditures, making bladder cancer the most expensive of all cancers [2], [3]. Thus, cost-effective prognostic strategies for evaluating incident and recurrent disease would be of significant clinical utility.

Epigenetic control of DNA expression is well known to drive fetal developmental differentiation. In a parallel fashion, in concert with genetic events (mutation, deletion and gene amplification) it is thought that epigenetic alterations may precipitate important pathological features of malignant degeneration [4]. Bladder cancer, with its divergent clinical (and pathological) phenotypes, presents a tumor model that may arise by inactivation of loci that independently control the propensity for invasion and, hence, dictate stage and grade, and that this inactivation may occur through a variety of epigenetic processes including microRNA alterations [5], alterations to chromatin [6], [7], and alterations to DNA methylation [8]. In this case, there is potential for the use of epigenetic alterations and particularly DNA CpG methylation as biomarkers for bladder cancers, as well as, potentially, for a variety of other human cancers [8], [9], [10], [11], [12], [13], [14]. Microarray-based approaches also have attempted to identify novel genes associated with invasive disease but with limited sample sizes due to the array strategy employed [15]. Recent developments in array approaches now allow for application of these technologies to population-based epidemiologic studies of cancer utilizing large numbers of samples [16], [17], [18]. There are numerous advantages to utilizing a population-based approach, including reduction in bias, greater generalizability of the results, and access to samples spanning all stages and grades of the tumor. Therefore, we have utilized this array-based approach to identify clinically and biologically informative patterns and novel gene targets of DNA CpG methylation in a population-based series of bladder transitional cell carcinoma.

## Results

### Identification of candidate loci

We utilized a 2-step approach to the identification and validation of loci that were associated with the development of invasive bladder cancer (Figure S1). In step 1, two independent series of tumors (Table 1) were analyzed by DNA methylation array to identify potential candidate loci having differential methylation, and in step 2, these candidates were confirmed in an additional series of tumors not profiled on the array.

**Table 1:** Demographics of the subject populations.

Characteristics	Series I – Profiled on Array (n = 73)	Series 2 – Profiled on Array (n = 264)	Series 3 – Used for Confirmation (n = 245)
**Age**, mean ±SD	62.1±9.3	65.1±9.9	62.9±9.1
**Gender**, n (%)			
Male	53 (73%)	200 (76%)	190 (78%)
Female	20 (27%)	64 (24%)	55 (22%)
**Invasive Stage Tumor**, n (%)*			
Non-invasive	42 (58%)	189 (72%)	181 (74%)
Low Grade (1–2)	35 (83%)	162 (86%)	158 (87%)
High Grade (3)	7 (17%)	27 (14%)	23 (13%)
Invasive	31 (42%)	75 (28%)	64 (26%)
**Tumor TP53 IHC Staining Intensity**, n (%)*			
Low (1,2)	50 (68%)	195 (74%)	189 (77%)
High (3+)	23 (32%)	69 (26%)	55 (23%)

*1 tumor in series 1 and one in the confirmation series did not have TP53 IHC staining data.

**Figure 1:**
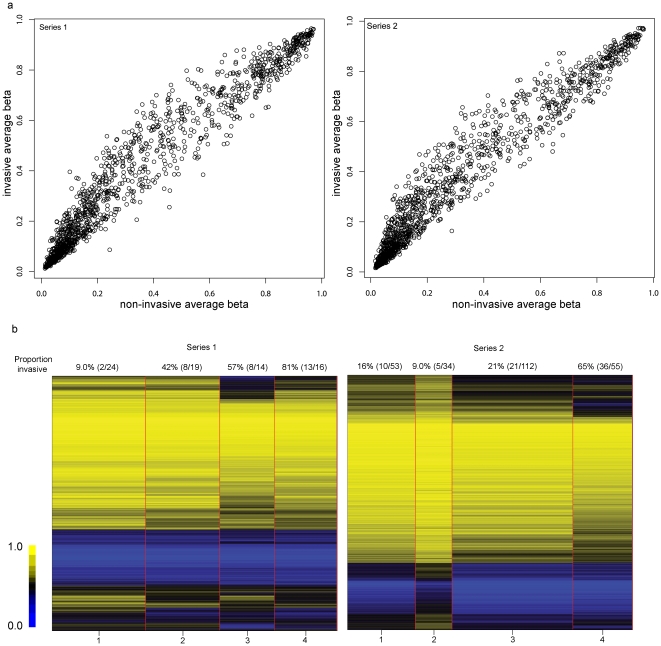
Methylation bead array profiles distinguish non-invasive and invasive bladder tumors. (A) Scatterplot of average methylation beta values in non-invasive bladder tumors (x-axis) and average methylation beta values in invasive tumors (y-axis) in series 1 (n = 73) and series 2 (n = 264) samples. (B) Recursive partition mixture models of each series result in 4 classes separated by red vertical lines, with the width of the classes corresponding to the number of samples in each class. Loci are represented as rows with the mean methylation for the class depicted. Above each class is the prevalence of invasive tumors (n invasive/total n) within the class. Methylation profiles significantly distinguish bladder tumor from non-diseased bladder epithelium (P<0.00001).

Overall, there was a general increase in methylation in invasive tumors (Figure 1A), suggesting that this approach has utility for demarcation of effective biomarkers. Generalized linear models comparing methylation levels of invasive versus non-invasive tumors at each locus confirmed this visual impression. In series 1, 445 CpG loci had significantly increased methylation in invasive compared to non-invasive tumors (*Q*<0.05) while only 68 loci had significantly decreased methylation. Similarly, in series 2, 606 loci had significantly increased methylation and only 41 had significantly decreased methylation (Table S1). Modeling each series, independently, with recursively partitioned mixture models yielded four methylation profiles (Fig 1B). Notably, significantly more invasive tumors were in methylation class 4 (P<0.00001, permutation chi-square).

To identify the loci most robustly associated with invasive disease, we utilized 3 distinct, commonly applied statistical approaches to identify those gene loci (from each of the three statistical approaches and across the 2 series) that overlapped in differentiating invasive and non-invasive disease. Five loci (*FRZB*_E186, *HOXB2*_P99, *KRT13*_P676, *RIPK1*_P868, *STAT5A*_P704) were found to be associated with invasive disease in all three statistical approaches.

For four of these loci (*FRZB*, *STAT5A*, *KRT13*, and *HOXB2*), bisulfite pyrosequencing assays were able to be designed, and were used to confirm the array findings in a subset of bladder tumors examined on the array. A pyrosequencing assay for *RIPK1* could not be successfully designed. For all CpG sites examined, as well as the mean across sites for *FRZB*, *KRT13*, and *HOXB2*, we observed significantly greater methylation in invasive compared to non-invasive tumors, consistent with the array results (Figure S2A, S2C, S2D). For *STAT5A*, we confirmed the significantly greater extent of methylation at the specific CpG site measured by the array, but did not observe this association in the neighboring CpG sites (Fig S2B), and so this loci was not further investigated.

### Treatment of a Bladder Cancer Cell Line with Methylation Inhibitor Leads to Re-expression of HOXB2

**Figure 2:**
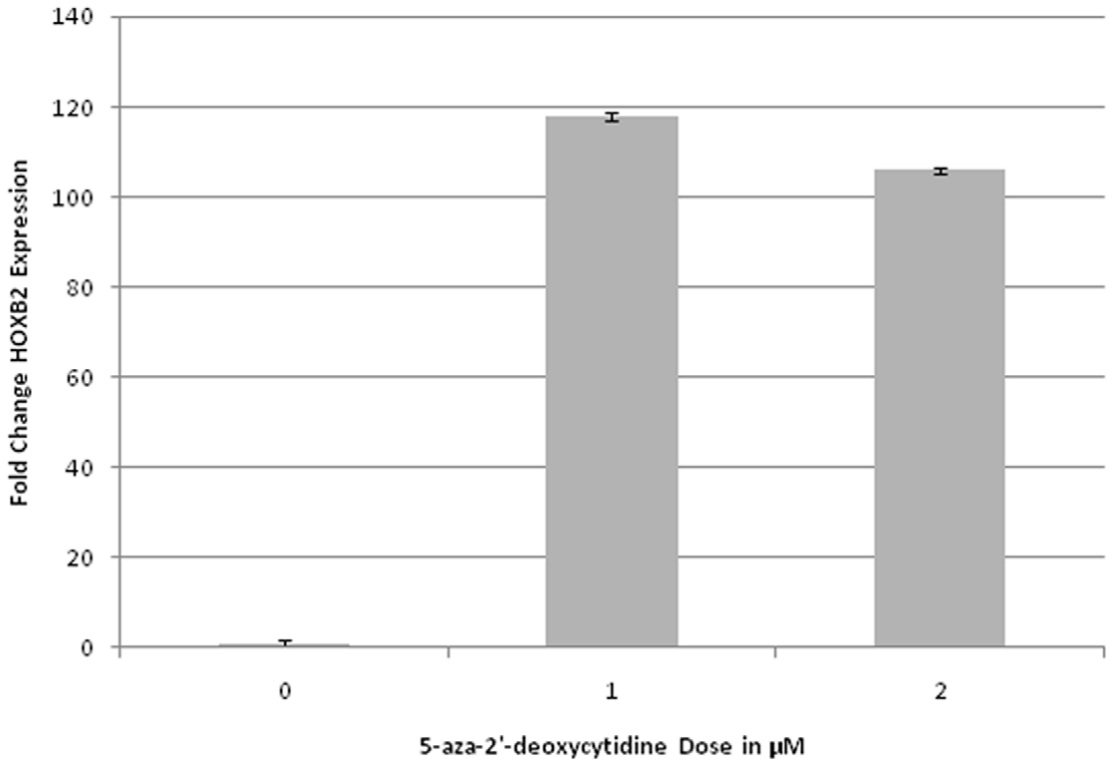
*HOXB2* is re-expressed following treatment of HTB-9 bladder cancer cells with 5-aza-2′deoxycytidine. Quantitative RT-PCR analysis was used to determine the gene expression of *HOXB2* in human bladder carcinoma cell line HTB-9, following a 5-day treatment with 1 or 2 µM 5-aza-2′-deoxycytidine or mock treatment. Bars represent the mean fold change in expression compared to mock treatment for 4 or 6 replicate experiments, and error bars denote the standard errors.

Pyrosequencing was performed to determine the methylation status of *HOXB2*, *FRZB*, and *KRT13* in bladder cancer cell lines HTB-9 and UM-UC3. HTB-9 had a HOXB2 methylation extent of 68.9, similar to what had been observed amongst primary bladder tumor samples, and thus was chosen for further examination. Treatment of HTB-9 cells with 1 or 2 µM 5-aza-2′-deoxycytidine led to an increase of greater than 100 fold expression of HOXB2 compared to mock-treated cells (Figure 2).

### HOXB2 Methylation is independently associated with invasive bladder cancer

**Figure 3:**
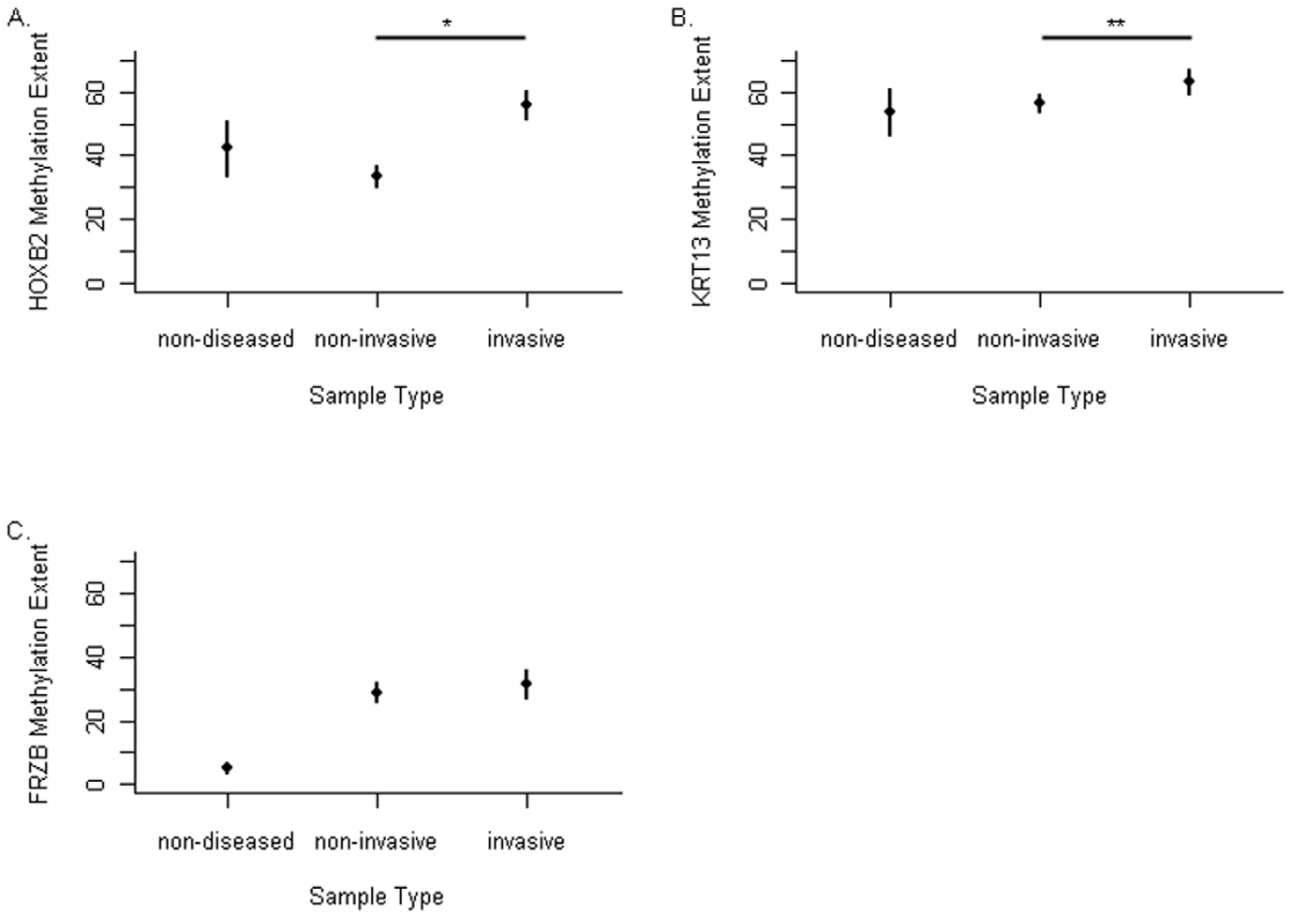
Bisulfite pyrosequencing reveals a greater extent of methylation in invasive bladder cancer. The mean (filled circle) and 95% confidence intervals of the extent of methylation of the promoter regions of (A) *FRZB*, (B) *KRT13*, and (C) *HOXB2* are depicted (y-axis) comparing non-diseased bladder epithelium, non-invasive tumors and invasive tumors (x-axis). The differences in the extent of methylation across these groups is significantly different (Kruskal-Wallis test), for *HOXB2* (P<0.00001) and *KRT13* (P<0.04), and *FRZB* (P<0.003). Comparisons specifically of noninvasive compared to invasive disease also revealed significant differences for *HOXB2* *P<0.00001 and *KRT13* **P<0.02.

Pyrosequencing of the promoter regions of *FRZB*, *KRT13*, and *HOXB2* was performed in an independent series of 263 bladder tumor patients not examined on the array as well as on 4 non-diseased tumor samples obtained from the NDRI. The extent of methylation at the pyrosequenced regions in non-diseased bladder epithelium, non-invasive tumors to invasive tumors is depicted on Figure 3, and reveals significant differences in methylation across the 3 categories for each of the genes (*HOXB2* P<0.00001, *KRT13* P<0.05, *FRZB* P<0.003, Kruskal Wallis Test). Methylation of *HOXB2* and *KRT13* are each significantly greater in invasive compared to non-invasive tumors (protected Wilcoxon rank sums test, P<0.00001 and P<0.02 respectively). TP53 immunohistochemical (IHC) staining intensity has been previously associated with a more aggressive disease [19], [20], and we included this variable when we performed logistic regression predicting invasive disease, for each of the loci individually, dichotomizing the methylation extent at the median, and controlling for other potential confounders. In models controlled for age, gender, and TP53 immunohistochemical staining intensity of the tumor, only *HOXB2* promoter methylation demonstrated an independent association with invasive tumors (Table 2). Tumors with *HOXB2* methylation had an OR of 7.7 (95% CI 3.3, 18.2) for being an invasive tumor. This result held up in a model controlling for all 3 loci as well as TP53 IHC staining intensity and patient age and gender, where *HOXB2* methylation was associated with an 8.6 fold increased risk of being an invasive tumor (95% CI 3.4, 21.7).

**Table 2:** Individual gene methylation associations with invasive bladder cancer.

	Non-invasive n (%)	Invasive n (%)	Invasive OR (95% CI)*	Non-invasive n (%)	Invasive n (%)	Invasive Disease OR (95% CI)*	Non-invasive n (%)	Invasive n (%)	Invasive Disease OR (95% CI)*	Non-invasive n (%)	Invasive n (%)	Invasive Disease OR (95% CI)†
**Total N**	171	61		170	62		177	61		162	57	
***HOXB2***												
Negative (≤median)	110 (93)	8 (7)	1.0 (referent)	–	–	–	–	–	–	104 (94)	7 (6)	1.0 (referent)
Positive (>median)	61 (54)	53 (46)	7.7 (3.3, 18.2)	–	–	–	–	–	–	58 (54)	50 (46)	8.6 (3.4, 21.7)
***KRT13***												
Negative (≤median)	–	–	–	91 (80)	22 (20)	1.0 (referent)	–	–	–	86 (82)	19 (18)	1.0 (referent)
Positive (>median)	–	–	–	79 (66)	40 (34)	1.3 (0.7, 2.7)	–	–	–	76 (67)	38 (33)	1.0 (0.4, 2.3)
***FRZB***												
Negative (≤median)	–	–	–	–	–	–	93 (80)	24 (20)	1.0 (referent)	84 (78)	23 (22)	1.0 (referent)
Positive (>median)	–	–	–	–	–	–	84 (69)	37 (31)	1.4 (0.7, 2.7)	78 (70)	34 (30)	0.9 (0.4, 2.0)
**TP53 IHC Staining Intensity**												
Low (<3)	154 (86)	26 (14)	1.0 (referent)	154 (86)	26 (14)	1.0 (referent)	160 (86)	26 (14)	1.0 (referent)	146 (85)	26 (15)	1.0 (referent)
High (3+)	17 (33)	35 (67)	7.4 (3.4, 16.0)	16 (31)	36 (69)	12.4 (5.9, 25.9)	17 (33)	35 (67)	12.1 (5.9, 24.9)	16 (34)	31 (66)	6.1 (2.7, 13.8)

*Model is adjusted for age, gender, and TP53 Staining Intensity.

† Model is adjusted for age, gender, each gene methylation variable, and TP53 Staining Intensity and includes only those subjects with data for all covariates.

### Methylation of HOXB2, FRZB, and KRT13 are Associated with Aggressive Non-invasive Bladder Cancer

**Figure 4:**
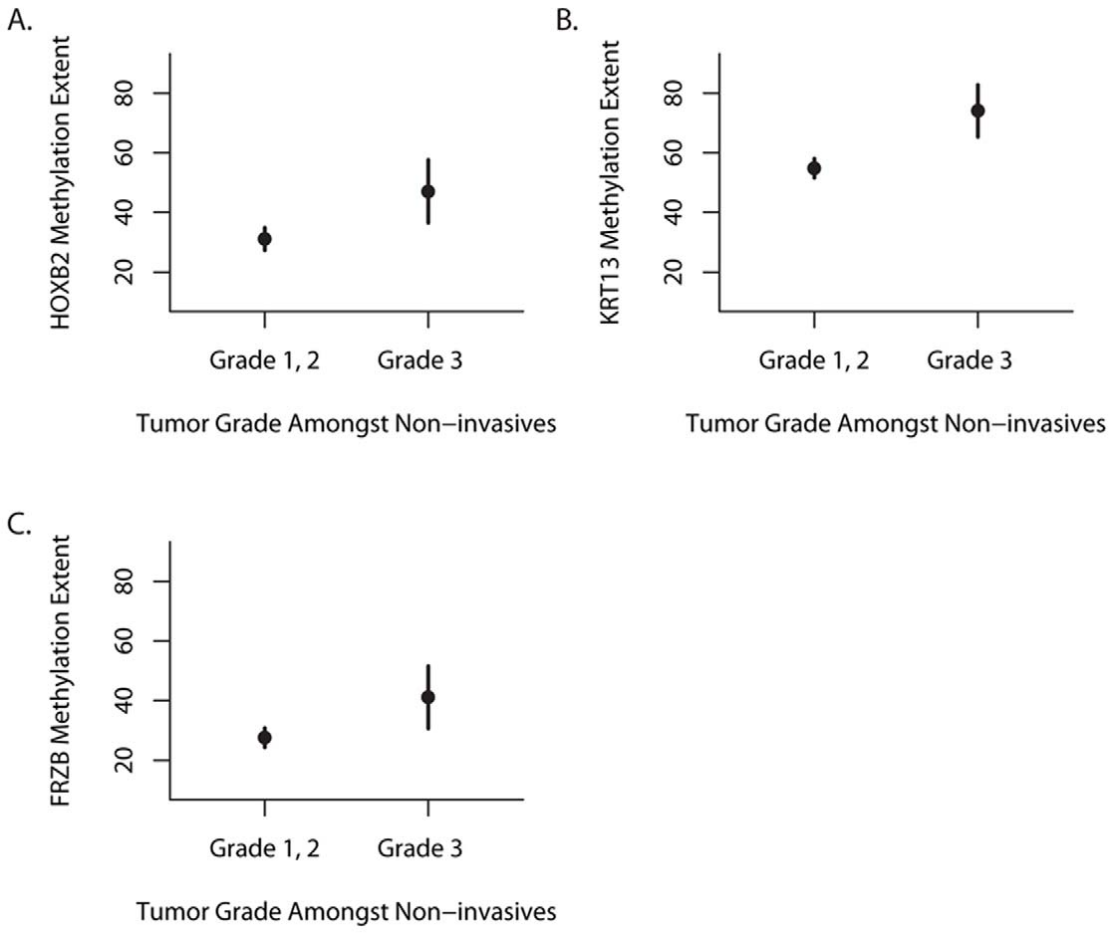
Bisulfite pyrosequencing demonstrates that extent of methylation is associated with tumor grade in non-invasive tumors. The mean (filled circle) and 95% confidence intervals of the extent of methylation of the promoter regions of (A) *FRZB*, (B) *KRT13*, and (C) *HOXB2* are depicted (y-axis) comparing low (1,2) to high (3+) grade tumors (x-axis). P-values resulting from the Wilcoxon Rank Sums demonstrate that these differences are significant for *HOXB2* (P<0.01), *KRT13* (P<0.01) and *FRZB* (P = 0.0001).

As we are interested in determining how methylation contributes to progression and aggressiveness of tumors, we examined the association between methylation extent and tumor grade within the non-invasive tumors. Figure 4 demonstrates that there is significantly greater extent of methylation of *HOXB2* (P<0.01), *KRT13* (P<0.01) and *KRT13* (P = 0.0001) in high grade (3) compared to low grade (1,2) non-invasive tumors. To control for potential confounding, we performed multivariable logistic regression analysis to examine the association between gene promoter methylation extent and tumor grade. Initially, we modeled each gene promoter individually, and observed significant associations between *FRZB* (OR 2.9, 95% CI 1.1, 7.9), and *KRT13* (OR 3.3, 95% CI 1.1, 10.1), but only a borderline significant association between *HOXB2* methylation and high tumor grade (OR 2.6, 95% CI 0.9, 6.9, Table S2). We then examined the additive effects of methylation of these 3 loci by modeling the association between methylation of all three loci compared to having none, 1, or 2 methylated and high-grade non-invasive disease (Table 3). Controlled for age, gender, and TP53 IHC staining intensity, methylation of all 3 loci was associated with a significant 7.4 fold increased risk of being a high grade tumor (95% CI 2.5, 22.1).

**Table 3:** Methylation of *HOXB2*, *FRZB* and *KRT13* is independently associated with high grade disease in non-invasive bladder cancer.

	Grade 1, 2	Grade 3	Grade 3 OR
	n(%)	n(%)	(95% CI)
Total N	143	19	
**HOXB2, FRZB, and KRT13 Methylation >Median**			
None, 1, or 2 Genes	125 (94)	8 (6.0)	1.0 (referent)
All 3 Genes	18 (62)	11 (38)	7.4 (2.5, 22.1)
**TP53 IHC Staining Intensity**			
Low (<3)	133 (91)	13 (9.0)	1.0 (referent)
High (3+)	10 (62)	6 (38)	4.7 (1.3, 17.4)

Each model is controlled for variables in columns, as well as age and gender.

## Discussion

Compared to gene-expression based markers, DNA methylation patterns are more stable and can be detected using various approaches requiring relatively small amounts of patient materials. In addition, these markers can be objectively identified and quantified, providing potential improvement to reliability in histological subjective diagnoses such as tumor grades or compared to immunohistochemical staining patterns which rely on individual interpretation of intensity and location of staining. For example, the markers identified and independently validated in this study hold promise as biomarkers useful in bladder cancer screening and follow-up for recurrence and progression and could be applied and tested for clinical utility in easy to collect urine sediments [9], [10], [11], [13], [21]. In addition, discovery of genes and pathways epigenetically altered in bladder tumors suggest novel targets for therapeutic intervention, especially when considering the poor prognosis associated with invasive bladder cancers. Identification of important and useful DNA methylation based markers is not without challenge, particularly as there is often a high degree of correlation between these alterations [22]. Therefore appropriate statistical approaches must be utilized to examine these alterations and to identify those alterations with the greatest clinical utility. We believe that our 2 stage approach provides for the most robust and generalizable identification of DNA methylation alterations which can be further examined in prospective studies. Using several different statistical approaches, our selection strategy for informative markers allowed us to identify a manageable number of potentially useful and robust biomarkers for laboratory validation as well as replication in independent samples. Most importantly, our validation of these markers in an independent group of patients provides compelling evidence of their potential utility and biological importance.

Previous studies examining panels of candidate genes have identified specific genes and panels of loci which are predictive of both the presence of tumor when examined in urine sediment [23] and the progression from non-invasive to invasive disease [15], [24], [25], [26] when examined within the tumor tissue. Specific genes such as *RASSF1A*, *RARB*, *BAMBI* and the *SFRP* family have been associated with higher grade and higher stage bladder cancer [8], [27], [28]. Our strategy did not identify these specific genes, and this discrepancy may be due to a number of differences between our approach and those previously utilized. We have made use of a population-based case series sample, while many previous studies have utilized hospital-based convenience series, and thus may have had some bias introduced in the samples examined. The array platform used in this study is limited in that it examines only a subset of all genes and only 1 or 2 specific CpG sites for those genes, and thus may miss the specific genes of loci previously described. Finally, our analytical approach, as it is based on the identification of panels of genes may identify different loci than those used in candidate analyses, but hypermethylation of those previously described candidates, may, in fact, be highly correlated to the methylation patterns identified herein. Further study combining previously identified loci with our own new targets in a prospective fashion is needed to more definitively determine those loci with the greatest clinical utility.

The biologic underpinnings of non-invasive yet recurrent disease versus invasive disease remain to be fully elucidated [29]. Our findings indicate that there are large differences in methylation between invasive and non-invasive tumors, with invasive tumors exhibiting a general increase in methylation compared to non-invasive tumors. As DNA methylation at any one CpG is a binary measure (either the cytosine is methylated or not) the difference in the degree of methylation may reflect potentially greater homogeneity in the selection of epigenetically altered cells in the development of invasive disease, a result consistent with our previous work examining only a limited panel of gene promoters in a single series of bladder tumors [14]. This greater degree of methylation may be driving the features of the invasive phenotype, or alternatively may be a consequence of selective pressures related to this phenotype. Although our approach in examining incident disease cannot fully distinguish these possibilities, it provides data illustrative of the urgent need for further examination of this epigenetic phenomenon.

Identification of specific genes whose DNA methylation pattern is associated with tumor phenotypes, including stage and grade also illuminates the particularly important biological processes and pathways required for genesis of these histologically defined tissue states. The *FRZB* gene (also known as *SFRP3*) is a member of the secreted frizzled receptor family of soluble proteins which binds to and antagonizes the WNT receptor. We and others have previously shown that hypermethylation of *SFRP* genes is strongly and significantly associated with invasive bladder cancer, confirming the importance of this pathway in this phenotype [8], [12]. Of the *SFRP* genes, only *SFRP1*, (in addition to *FRZB*) is profiled on the array, and at a different CpG location than the primers used in our and previous reports of its methylation [8], [30]. This may explain why this loci was not identified in our screening approach. Additional, more genomically dense investigation of the WNT pathway is warranted, as this may suggest a novel route for therapeutic intervention for this disease.

*HOXB2* is a member of the homeobox family of transcription factors, and is encoded on chromosome 17 as part of a gene cluster with other HOX family members. Interestingly, we observe that non-diseased bladder epithelium exhibits a similar extent of methylation at *HOXB2* and *KRT13* as the non-invasive disease, while at *FRZB* the non-diseased tissue has a significantly lower extent of methylation than either of the 2 tumor types. We also observe, in the human bladder carcinoma cell line HTB-9, which demonstrates an extent of methylation similar to that observed in invasive tumors, that treatment with the methylation inhibitor 5-aza-2′-deoxycytidine leads to increased expression of *HOXB2*, suggesting that methylation of this gene may be functionally leading to the inactivation of this gene. The results in *HOXB2* are consistent with the literature which has described a bivalent domain structure of the chromatin of homeobox genes, wherein both pluripotent and terminally differentiated tissue exhibit marks of both active and repressive chromatin within the same region [31], [32], and thus an intermediate extent of DNA methylation. Invasive bladder cancers appear targeted for hypermethylation of this region, a phenomenon previously reported across polycomb group controlled genes in colorectal cancer cells [33]. Such a differential alteration of a key developmental gene may be critical in defining the phenotypes of these tumors and explain the behavior and outcome from these disparate forms of this disease.

*KRT13* encodes cytokeratin-13 which has been shown to have specific expression in cervical squamous tumors and in mucinous cervical type adenocarcinomas [34]; to our knowledge, epigenetic alterations to this gene have not been reported. Epigenetic alteration of this gene may affect its expression pattern, and such changes may be signs of a more de-differentiated phenotype, considered characteristic of aggressive, high grade tumors.

We demonstrated in an independent confirmation series of tumors that the extent of methylation of *HOXB2* and *FRZB* is associated with tumor grade, independent of each other and of TP53 protein immunohistochemical staining. TP53 staining has previously been examined and has been touted as a useful prognostic marker in this disease [35], [36], [37], although there is conflicting data on its utility [36], [38], [39], [40]. A recent meta-analysis examining its utility suggests that there is not appropriate evidence to suggest utilizing this marker clinically [20], as it may provide no addition prognostic information other than a strong association with the invasive phenotype of the disease [41], [42]. We cannot yet suggest that the markers discovered using our approach hold any more clinical utility than pathological grading and staging of tumors, but we believe that our data suggest specific cellular pathways that are disrupted in this disease. Thus, these pathways could be explored intensively as novel targets for therapeutic strategies. In addition, we suggest that future work, examining the utility of these markers in a prospective fashion is needed, as they may be useful in determining which tumors may progress. Indeed, these markers might also contribute to clinical efforts to follow patients with less invasive methods, to determine if their tumor has recurred or progressed, thereby requiring more aggressive treatment approaches.

## Methods

### Study Population and Sample Ascertainment

All study participants provided written informed consent under the approval of the institutional review boards of Dartmouth Medical School and Brown University. We utilized two, independent, non-consecutive population-based series of bladder cancer cases. The first, consisting of tumors from 344 individuals involved in a case-control study of incident bladder cancer in New Hampshire, diagnosed between July 1994 and June 1998 [43], and the second consisting of tumors from 264 individuals diagnosed between January 1, 2002 to July 30, 2004 [44]. Although separate in time and scope, these two studies utilized identical recruitment procedures and study personnel as well as identical protocols for the ascertainment of pathology materials for molecular examinations. Bladder tumors from both series were reviewed by the study pathologist (A.R.S.) and classified according to the 1973 and 2004 World Health Organization guidelines for bladder tumors. The study pathologist identified the appropriate block from which the tumor samples used in these analyses were obtained, and the proportion of malignant cells in each sample was estimated. All samples used in the examination contained >75% tumor sample. Carcinoma in-situ was excluded from analysis due to limited sample size. Table 1 describes the characteristics of the subjects included in the final analysis. In addition, four non-diseased bladder epithelium samples were obtained from the National Disease Research Interchange (NDRI) all from autopsy specimens of individuals who did not have a diagnosis of cancer.

### DNA Extraction and Methylation Analysis

Tumor sections with the greatest proportion of malignant tissue were selected by the study pathologist for use in our molecular analyses. DNA was extracted and sodium bisulfite modified following standard procedures as described in Marsit, et al. [18]. A total of 82 tumors from the first series and all 264 tumors from the second series were profiled for the methylation status at 1505 CpG loci using the Illumina GoldenGate® methylation bead arrays. Bead arrays were run at the UCSF Institute for Human Genetics, Genomics Core Facility according to the manufacturer's protocol and as described in Bibikova, et al. [45].

### Statistical analysis

We assembled data with BeadStudio Methylation software from the array manufacturer (Illumina, San Diego, CA) and quality assessment performed as in Christensen et al. [16] resulting in seven samples (9%), where >75% of loci had a detection p-value >1*10^−5^, being dropped from analysis. A similar quality control for CpG loci eliminated those loci with median detection p-value >0.05 (n = 8, 0.5%).

Subsequent analyses were carried out in The R Package. For exploratory/visualization purposes, hierarchical clustering using the Manhattan metric and average linkage was performed. For locus by locus analyses, associations at individual CpG loci were tested with a generalized linear model (GLM). Under the assumption that the average beta values follow a beta distribution [46], a quasi-binomial model (logit link, binomial variance, and non-unit scale parameter) was used; note that the logit link function imposes an appropriate constraint on the mean average beta. To adjust for multiple comparisons in scanning the 1413 autosomal CpG loci, P-values for associations between average beta and invasive disease used false discovery rate correction and q-values computed by the *qvalue* package in R. An FDR q-value of <0.05 was considered significant for this examination.

*Random Forests* [47](http://www.math.usu.edu/~adele/forests/),was used to build classifiers of invasive stage (versus noninvasive) by CpG average beta values. Analysis was conducted in R using the *randomForest* package (version 4.5-18) by Liaw and Wiener. At each node of the tree, a random sample of *m* out of the total *M* variables was chosen and the best split is found among the *m* variables. The default value for *m* in the Random Forest R package is 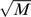. In this analysis we tested a range of *m* from half of 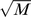 to two times 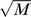 and used *m* that gave the lowest prediction error; in this case *m* = 38. The OOB error rate is the percentage of time the RF prediction is incorrect. A test for association between methylation (predictors) and sample type was conducted by comparing the OOB obtained on the data set with the null distribution of OOB errors obtained by permuting sample type labels and running the RF procedure 100 times. We also used the second series of 264 tumors as a validation set, computing the error rate as each tree is constructed, to obtain an estimated error rate independent of the first series. Finally, we used the variable importance scores, percent change in mean squared error (MSE), to identify loci that had the greatest influence on classification, by choosing those loci whose percent change in MSE was greater than 5%.

For inference, data were clustered using a mixture model [48] with a mixture of beta distributions [49], and the number of classes was determined by Bayesian information criterion (BIC) [50], [51]. The mixture model was fit by recursively partitioning the data using a 2-class mixture model, with a variant of BIC used as a criterion for the split, as described in [46]. Class membership was obtained from the mixture model using an empirical Bayes procedure, and subsequent associations with invasive stage were tested via permutation test with 10,000 permutations each using the standard chi-square goodness-of-fit test. The output of mixture models includes a class-specific beta distribution *F_jk_* for each locus *j* and class *k*. These distributions imply a receiver operating curve (ROC) for distinguishing two classes *k* and *l* by the relation 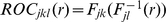 or 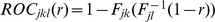, whichever curve lies above the identity line. In either case, the area-under-the-ROC (AUC), computed as 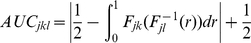, which can be used to determine the influence of locus *j* on the distinction between classes *k* and *l*. A similar procedure can be used to distinguish class *k* from the others: we use a normal approximation *G_jk_* for the distribution of the combined classes other than *k*, and compute 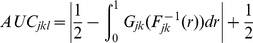. We then ranked each locus j by its computed AUC_jk_, and selected those with AUC>0.90.

To identify loci for subsequent validation as predictors of invasive vs. non-invasive disease, we computed the intersection of the loci that were most significant (Q<0.05) in the locus-by-locus analysis, the top ranking loci from the RF analysis (% change in MSE>6%), and the loci having AUC>0.75 for distinguishing the class of interest compared to others in each tumor series, and then identified those loci overlapping in both series.

### Bisulfite Pyrosequencing

Quantification of cytosine percent methylation was performed by pyrosequencing bisulfite-converted DNA using the PyroMark MD pyrosequencing system (Qiagen, Valencia, CA). Specific pyrosequencing primers were designed to amplify array CpG sites and as many downstream CpGs as conditions permitted (1 to 5 additional) using Biotage Assay Design Software v1.0.6 (Qiagen). Assays designs were attempted for all 5 of the loci identified as overlapping from the 3 analysis strategies and assays were successfully designed for 4 of the loci. Primer details and the genomic location of the region sequenced relative to the transcription start site are provided in Table S3. The sequences used were based on the reference sequence assembly 36.1. All PCR reactions, performed using Qiagen Hot Star Taq polymerase, included a no template control, unmodified DNA control, and 7 standardized percent methylation controls (0%, 15%, 25%, 45%, 65%, 75%, and 100%) derived from Qiagen EpiTect® PCR control DNA set samples. Sequencing reactions used 10µl of PCR product and were run according to instrument/manufacturer protocols (Biotage).

### Statistical Analysis of Pyrosequencing Data

Associations between pyrosequencing percent methylation values at individual CpG sites were examined using the Spearman rank correlation. The mean value of CpG methylation across the examined CpG sites was used in further analyses. To examine the association between methylation extent as a continuous variable and tumor grade, we utilized the non-parametric Kruskal-Wallis one-way analysis of variance. To adjust for potential confounders and to determine the odds ratio (OR) and 95% confidence interval (CI) for high grade disease, we used multivariable unconditional logistic regression analysis to examine the association between methylation extent (in quartiles) at individual sites and tumor grade (grade 1 vs. grade 2, 3, 4), with adjustment for potential confounders. We also included a term to control for percent of tumor cells within sample in the model, and found that there were no significant changes in the effect estimates or significance of the main effects, and thus this term was not considered a confounder as was removed from the model.

### Cell Culture and Drug Treatment

Human bladder carcinoma cell lines UMUC3 and HTB-9 were purchased from the American Type Culture Collection (Manassas, VA). Cell lines were grown in their respective vendor-recommended culture media at 37°C and 5% CO_2_ and passaged every 3–5 days. Approximately 1 million cells were harvested from each and DNA isolated and sodium bisulfite modified as above to examine methylation status of the candidate loci. 5-aza-2′-deoxycytidine was obtained from Sigma-Aldrich (St. Louis, MO), and was prepared in sterile DMSO. The concentrations used for the experiment were chosen to mimic those observed clinically in human plasma [52] and that did not result in significant loss in cell viability. For experiments, cells were seeded 24 hours before drug treatment, 20,000 cells per well into a 6-well cell culture plate. The media was replaced with that containing 5-aza-2′-deoxycytidine, and was changed on day 1 and 3. Cells were harvested on day 5 and immediately subjected to RNA isolation.

### RNA Isolation and Quantitative RT-PCR Analysis

Total RNA was isolated from cells using the Qiagen RNEasy RNA isolation system, following manufacturer's protocols, and including an on-column DNAse digestion. The cDNA was prepared from 150 ng total RNA using the Taqman Gene Expression system (Applied Biosystems, Carlsbad, CA). cDNA, diluted 1∶5 was subjected to RT-PCR for HOXB2 (Hs01911157_s1) and ACTB (hu ACTB) as the referent gene using pre-designed Taqman primers and probes on an ABI 7500 Fast Real Time PCR system (Applied Biosystems). All RT-PCRs were run in triplicate and no-template and no reverse transcription controls were included for each assay. Fold change was calculated using the Pfaffl method [53].

## Supporting Information

Figure S1 Diagram of the methodology used in the selection of loci for follow-up analyses and validation.
(0.27 MB TIF)

Figure S2 Confirmation of DNA methylation of FRZB (A), STAT5A (B), KRT13 (C), and HOXB2 (D) by pyrosequencing in samples analyzed on the array. Dots represent percent methylation at each position examined (POS) in non-invasive (N) compared to invasive (I) tumor tissue. The gray box encloses the position which represents that position examined on the array. *P<0.05, **P<0.0001.
(1.68 MB TIF)

Table S1 Results of locus by locus analysis comparing invasive to non-invasive bladder cancers with Q<0.001.
(0.56 MB PDF)

Table S2 Individual gene promoter methylation is associated with high grade in non-invasive bladder cancer.
(0.05 MB PDF)

Table S3 Primer sequences used for bisulfite pyrosequencing reactions.
(0.08 MB PDF)
