# Supplementary figures and images for: Identification of Methylated Genes Associated with Aggressive Bladder Cancer
Source: PLoS One. 2010 Aug 23;5(8):e12334. doi: 10.1371/journal.pone.0012334 (PMC2925945; doi:10.1371/journal.pone.0012334)

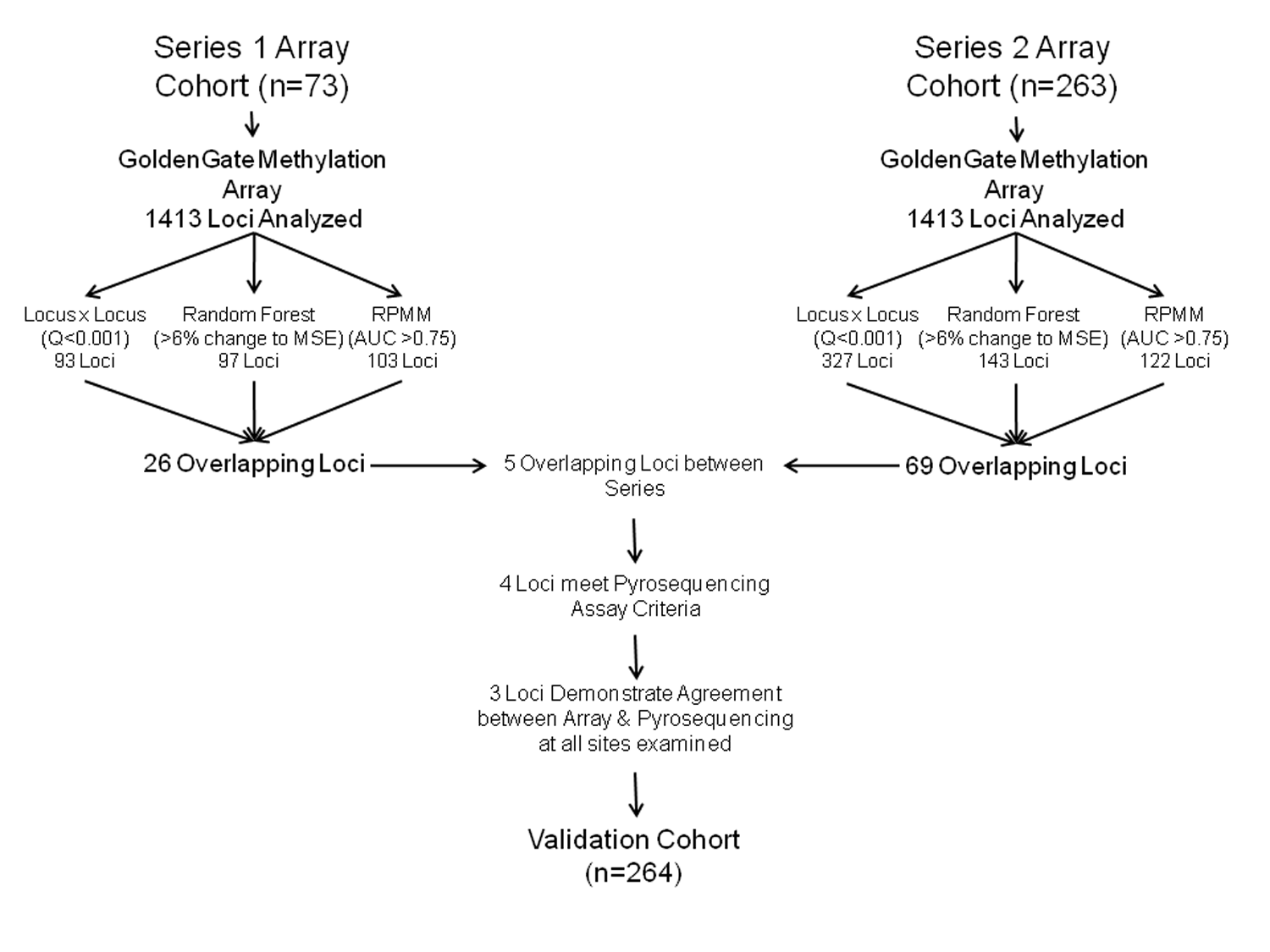

Supplement: Figure S1 — Diagram of the methodology used in the selection of loci for follow-up analyses and validation. (0.27 MB TIF) [file pone.0012334.s001.tif]
