# Supplementary material for: Identification of Methylated Genes Associated with Aggressive Bladder Cancer
Source: PLoS One. 2010 Aug 23;5(8):e12334. doi: 10.1371/journal.pone.0012334 (PMC2925945; doi:10.1371/journal.pone.0012334)
Supplement: Table S1 — Results of locus by locus analysis comparing invasive to non-invasive bladder cancers with Q<0.001. (0.56 MB PDF) [file pone.0012334.s003.pdf]

Table S1. Results of locus by locus analysis comparing invasive to non-invasive bladder cancers with Q<0.001.

Series 1

Label	coefficient	P-value	Q-value
SLC14A1_E295_F	1.3387325	0	0
RARA_P1076_R	1.41936497	0	1.00E-06
EGF_P413_F	1.08910466	0	1.00E-06
KRT13_P341_R	1.21180792	0	1.00E-06
CSF1R_P73_F	0.91514094	0	1.00E-06
FGFR4_P610_F	0.88923859	0	2.00E-06
UGT1A1_P315_R	1.04155841	0	3.00E-06
STAT5A_P704_R	1.2174913	0	4.00E-06
MAP3K1_P7_F	0.5168024	0	5.00E-06
IRF5_P123_F	0.3463398	0	6.00E-06
RIPK1_P868_F	0.83401194	0	6.00E-06
CSF1R_E26_F	1.00622584	0	7.00E-06
TEK_E75_F	1.09336758	0	7.00E-06
FGF1_P357_R	1.00168074	0	7.00E-06
TRPM5_P979_F	1.20272759	0	7.00E-06
SNCG_E119_F	1.04509079	0	1.00E-05
AATK_E63_R	1.2549204	1.00E-06	1.80E-05
SLC14A1_P369_R	1.14971128	1.00E-06	1.90E-05
FGF1_E5_F	0.9061877	1.00E-06	2.40E-05
CSF2_P605_F	1.07409814	1.00E-06	2.50E-05
HPN_P374_R	1.0980553	1.00E-06	3.00E-05
CSF3_P309_R	1.09172212	1.00E-06	3.40E-05
TMPRSS4_E83_F	0.9931844	2.00E-06	5.20E-05
AIM2_E208_F	0.5955002	3.00E-06	6.60E-05
AATK_P519_R	1.06967047	3.00E-06	6.80E-05
CDH17_P376_F	0.95001407	4.00E-06	7.30E-05
MMP7_E59_F	0.62120749	4.00E-06	8.00E-05
THBS2_P605_R	0.8676801	4.00E-06	8.00E-05
KLK10_P268_R	0.46714679	6.00E-06	0.000113
TFF2_P178_F	0.95835135	9.00E-06	0.000163
CLDN4_P1120_R	0.81908248	9.00E-06	0.000164

Series 2

Label	coefficient	P-value	Q-value
GP1BB_P278_R	1.3856323	0	0
SLIT2_E111_R	1.1433043	0	0
FGF3_E198_R	1.553377	0	0
STAT5A_P704_R	1.1463442	0	0
CDH11_E102_R	1.5658691	0	0
KRT13_P341_R	1.0192842	0	0
FGF1_P357_R	0.924289	0	0
SLIT2_P208_F	1.3797546	0	0
EYA4_E277_F	1.2962476	0	0
RASSF1_E116_F	1.6330738	0	0
HOXB2_P488_R	1.2223127	0	0
CSF3_P309_R	0.9188403	0	0
CDH13_E102_F	1.2863378	0	0
HPN_P374_R	1.0339484	0	0
TNFRSF10C_P7_F	1.0423888	0	0
TERT_P360_R	0.9213992	0	0
HS3ST2_P171_F	1.253037	0	0
SOX1_P294_F	1.1985183	0	0
TPEF_seq_44_S36_F	1.1313103	0	0
NTSR1_P318_F	1.2125521	0	0
DES_E228_R	1.0163863	0	0
NOTCH4_E4_F	1.0598946	0	0
FGF3_P171_R	1.2871834	0	0
GDF10_P95_R	1.186943	0	0
VIM_P343_R	1.2593558	0	0
MST1R_E42_R	1.0085818	0	0
GAS7_P622_R	1.1731587	0	0
VAV1_E9_F	1.0766027	0	0
RASSF1_P244_F	1.531879	0	0
TNFRSF10C_E109_F	1.0598858	0	0
AGTR1_P41_F	1.3044499	0	0

JAG2_P264_F	0.35467631	1.10E-05	0.000183	IRAK3_P13_F	1.4429374	0	0
MMP19_E274_R	0.7508523	1.20E-05	0.000198	TEK_E75_F	0.958363	0	0
KRT13_P676_F	1.13377858	1.30E-05	0.000214	RIPK1_P868_F	1.1594791	0	0
MDR1_seq_42_S300	1.76354384	1.60E-05	0.000218	CDH13_P88_F	1.064542	0	0
NOS2A_P288_R	0.76216239	1.60E-05	0.000218	THY1_P149_R	0.9152907	0	0
CLK1_P538_F	0.633496	1.60E-05	0.000218	CHGA_E52_F	1.0041443	0	0
ER_seq_a1_S60_F	0.63568692	1.60E-05	0.000218	FRZB_E186_R	1.2636902	0	0
KRT5_P308_F	0.80840704	1.60E-05	0.000218	AGTR1_P154_F	1.1014844	0	0
CPA4_E20_F	0.80337942	1.60E-05	0.000218	EYA4_P794_F	1.1116137	0	0
P2RX7_P597_F	0.97233782	1.80E-05	0.00024	NPY_E31_R	1.051733	0	0
IRF7_E236_R	0.99565263	2.10E-05	0.000268	CDH11_P203_R	1.2740182	0	0
DUSP4_E61_F	0.42452417	2.20E-05	0.000274	HHIP_E94_F	1.1108807	0	0
SFTPA1_E340_R	0.95824726	2.20E-05	0.000274	GALR1_E52_F	1.1923132	0	0
HPN_P823_F	0.97543934	2.50E-05	0.000284	SFRP1_P157_F	1.2512258	0	0
HOXB2_P99_F	1.2983317	2.50E-05	0.000284	NPY_P295_F	1.2095289	0	0
TRIM29_E189_F	0.76405834	2.50E-05	0.000284	TMEFF2_E94_R	1.0946249	0	0
ACVR1C_P115_R	0.41361271	2.50E-05	0.000284	SFRP1_E398_R	1.5638553	0	0
CXCL9_E268_R	0.79921555	2.60E-05	0.000284	HOXB2_P99_F	1.0934193	0	0
HOXA5_E187_F	1.16793045	2.90E-05	0.000306	RARA_P1076_R	0.8414265	0	0
JAK3_P1075_R	0.73680387	2.90E-05	0.000306	DBC1_E204_F	1.0727138	0	0
FRZB_E186_R	1.15935589	3.20E-05	0.000327	PADI4_P1158_R	0.7860577	0	0
TUSC3_E29_R	0.89233563	3.20E-05	0.000327	NRG1_P558_R	1.1835839	0	0
PSCA_P135_F	0.75537542	3.30E-05	0.000329	PYCARD_P150_F	0.9018168	0	0
TFF1_P180_R	0.76488495	3.50E-05	0.000342	SLC5A8_E60_R	0.9634903	0	0
TRIM29_P135_F	0.7005761	3.80E-05	0.000365	MOS_E60_R	1.0888752	0	0
NBL1_P24_F	0.75957498	3.90E-05	0.000372	TERT_E20_F	1.0318854	0	0
RARA_P176_R	0.89914696	4.10E-05	0.000386	EYA4_P508_F	1.0240425	0	0
UGT1A1_E11_F	1.17495539	4.30E-05	0.000394	S100A4_E315_F	0.9183224	0	0
TSC2_E140_F	0.80091218	4.90E-05	0.000446	EPHA5_E158_R	1.0737941	0	0
CDH17_E31_F	0.91226843	5.20E-05	0.000459	TAL1_E122_F	1.113453	0	0
SERPINB5_P19_R	0.85714216	5.30E-05	0.000459	SMO_E57_F	1.2898326	0	0
MBD2_P233_F	0.54766266	5.70E-05	0.000487	ISL1_E87_R	1.0906623	0	0
BMP2_P1201_F	0.70842245	5.80E-05	0.000493	LY6G6E_P45_R	0.8222785	0	0
SIN3B_P607_F	-0.6672309	5.90E-05	0.000493	PALM2-AKAP2_P18	0.8753533	0	0

LY6G6E_P45_R	0.88310234	6.20E-05	0.000506	IRAK3_E130_F	1.2832149	0	0
CSF2_E248_R	0.81282258	6.40E-05	0.000506	TPEF_seq_44_S88_F	0.9681109	0	0
USP29_P282_R	-0.3407678	6.40E-05	0.000506	MST1R_P392_F	1.0423856	0	0
SYK_P584_F	0.57231428	6.50E-05	0.000506	ETV1_P235_F	1.0055813	0	0
GPR116_E328_R	0.75472759	7.00E-05	0.000539	FLI1_P620_R	0.9731102	0	1.00E-06
SRC_P164_F	0.72024446	7.30E-05	0.000559	FGF1_E5_F	0.7999276	0	1.00E-06
FRK_P258_F	0.79500825	7.80E-05	0.000579	NTSR1_E109_F	1.277895	0	1.00E-06
PTPN6_P282_R	0.66121555	7.80E-05	0.000579	HS3ST2_E145_R	0.9275806	0	1.00E-06
DDIT3_P1313_R	1.06900865	8.10E-05	0.000594	DIO3_P90_F	0.6541632	0	1.00E-06
SRC_E100_R	0.83428926	8.60E-05	0.000622	HTR1B_P222_F	1.2354746	0	1.00E-06
CALCA_P171_F	0.68635294	9.00E-05	0.000637	AATK_E63_R	0.8591448	0	1.00E-06
PTHR1_P258_F	0.57101351	9.30E-05	0.000654	GALR1_P80_F	1.0666438	0	1.00E-06
PCDH1_E22_F	0.15375524	9.50E-05	0.00066	TMEFF2_P152_R	0.9267151	0	1.00E-06
SPDEF_E116_R	0.74037902	9.60E-05	0.00066	STAT5A_E42_F	0.886192	0	1.00E-06
NOS2A_E117_R	0.72944992	1.00E-04	0.000675	ISL1_P379_F	1.0035576	0	1.00E-06
MUC1_E18_R	0.54943113	0.000105	0.000702	IRAK3_P185_F	1.0455466	0	1.00E-06
ALOX12_E85_R	1.04007998	0.000114	0.00075	DLK1_E227_R	1.069632	0	1.00E-06
TRIM29_P261_F	0.91859733	0.000117	0.000764	CSPG2_P82_R	0.7855588	0	1.00E-06
FRK_P36_F	0.83581872	0.00012	0.000775	FGFR4_P610_F	0.7413949	0	1.00E-06
NRG1_P558_R	1.44089082	0.000126	0.000803	POMC_P400_R	1.070896	0	1.00E-06
HOXA5_P479_F	0.72240048	0.000128	0.000804	FLT4_E206_F	0.9297426	0	1.00E-06
ACVR1_P983_F	0.80187869	0.000131	0.000813	FLI1_E29_F	0.9040784	0	1.00E-06
HS3ST2_P171_F	1.20267811	0.000143	0.000879	THY1_P20_R	0.6663777	0	1.00E-06
IGFBP1_P12_R	0.84766875	0.000145	0.000881	SEPT9_P374_F	0.8974967	0	1.00E-06
ESR1_E298_R	0.70043802	0.000147	0.000882	EGF_P413_F	0.7077107	0	1.00E-06
SNCG_P53_F	0.84612824	0.000149	0.000884	CDH11_P354_R	0.8937976	0	1.00E-06
IGFBP1_E48_R	0.72242771	0.000153	9.00E-04	PDGFRB_P343_F	1.1876677	0	1.00E-06
TGFB3_E58_R	0.84300365	0.000171	0.000994	NOS2A_P288_R	0.7249621	0	2.00E-06
IGSF4_P86_R	1.08145882	0.000177	0.001019	MST1R_P87_R	0.8338096	0	2.00E-06
RHOH_P121_F	-0.5470529	0.000192	0.001091	PENK_E26_F	0.8019755	0	2.00E-06
ACVR1_E328_R	1.09573254	0.000201	0.001127	FES_P223_R	1.0942151	0	2.00E-06
IL1RN_E42_F	0.72121815	0.000202	0.001127	HS3ST2_P546_F	0.8358811	0	2.00E-06
HLA-DOB_E432_R	0.49977971	0.000208	0.001149	DCC_P471_R	0.9159515	0	2.00E-06
BCR_P346_F	0.80134025	0.000228	0.001246	FRZB_P406_F	1.3054302	0	2.00E-06

GABRG3_P75_F	-0.8129993	0.000231	0.001252
SEPT5_P464_R	0.80528153	0.00024	0.001283
IGF1R_P325_R	0.17419697	0.000248	0.001302
DAPK1_E46_R	0.6147292	0.000248	0.001302
NBL1_E205_R	0.71030449	0.00026	0.001346
PSCA_E359_F	0.74936903	0.000262	0.001346
PTPRO_P371_F	0.96029149	0.000264	0.001346
VAV1_P317_F	0.71296842	0.00027	0.001362
CHI3L2_E10_F	-0.6407692	0.000278	0.001393
CSPG2_P82_R	0.93818619	0.000295	0.00145
MOS_E60_R	1.0984238	0.000295	0.00145
MMP10_E136_R	0.69339667	0.000303	0.001478
EYA4_E277_F	1.03689085	0.000316	0.001524
NOTCH4_E4_F	0.94434317	0.000322	0.001541
WNT8B_P216_R	-0.3139461	0.000331	0.001556
AATK_P709_R	0.79532088	0.000331	0.001556
SFN_E118_F	0.66917429	0.000336	0.001564
PLAT_P80_F	0.77139259	0.00034	0.001569
VAMP8_P241_F	0.56239139	0.000357	0.001635
CEACAM1_P44_R	0.49917	0.000372	0.001691
NEFL_P209_R	0.97457343	0.000378	0.001704
COL18A1_P365_R	0.70828176	0.000384	0.001716
SEPT5_P441_F	0.86004162	0.000387	0.001716
GJB2_P931_R	0.94076309	0.00039	0.001716
GUCY2D_E419_R	1.23606679	0.00041	0.001786
TERT_P360_R	0.81567657	0.000423	0.001822
ZIM2_P22_F	0.57899961	0.000425	0.001822
PRSS8_E134_R	0.63871692	0.000441	0.001879
NOS3_P38_F	0.64751878	0.000448	0.001882
BCR_P422_F	0.81497796	0.000451	0.001882
PLAT_E158_F	0.72835928	0.000453	0.001882
MAPK10_E26_F	0.93485879	0.00046	0.001897
HS3ST2_P546_F	0.82367736	0.000478	0.00196
TRPM5_P721_F	0.6417481	0.000483	0.001962

IGF2_P1036_R	0.8127754	0	2.00E-06
MMP2_P197_F	0.7507948	0	2.00E-06
CSF2_P605_F	0.8779001	0	2.00E-06
WT1_E32_F	1.1121682	0	2.00E-06
BCR_P422_F	0.8020271	0	2.00E-06
IGFBP1_P12_R	0.872092	1.00E-06	3.00E-06
MT1A_P49_R	1.2656977	1.00E-06	3.00E-06
FABP3_P598_F	0.6937578	1.00E-06	3.00E-06
PRKCDBP_E206_F	1.0775421	1.00E-06	3.00E-06
JAK3_E64_F	0.9228389	1.00E-06	3.00E-06
UGT1A1_E11_F	0.920334	1.00E-06	3.00E-06
TBX1_P520_F	0.6994578	1.00E-06	3.00E-06
IPF1_P750_F	1.07221	1.00E-06	4.00E-06
KRT13_P676_F	0.9459594	1.00E-06	4.00E-06
CD9_P504_F	0.6695146	1.00E-06	4.00E-06
FLT1_P615_R	1.0460489	1.00E-06	5.00E-06
HCK_P46_R	0.9954437	1.00E-06	5.00E-06
PTPRG_P476_F	1.2027011	1.00E-06	5.00E-06
OPCML_E219_R	1.0584322	1.00E-06	5.00E-06
SLC5A5_E60_F	0.7329972	1.00E-06	5.00E-06
CSF3R_P8_F	0.7655531	1.00E-06	5.00E-06
ASCL2_E76_R	0.9163852	1.00E-06	5.00E-06
BMP4_P199_R	0.7020417	1.00E-06	5.00E-06
OPCML_P71_F	0.7024595	1.00E-06	5.00E-06
SPP1_E140_R	0.6648585	1.00E-06	5.00E-06
ACVR1_E328_R	0.847399	1.00E-06	5.00E-06
MT1A_E13_R	0.8307517	1.00E-06	5.00E-06
COL1A2_P48_R	1.0694814	1.00E-06	5.00E-06
WNT10B_P993_F	0.9040558	1.00E-06	6.00E-06
VAV1_P317_F	0.7398423	1.00E-06	6.00E-06
CD9_P585_R	0.716933	2.00E-06	6.00E-06
WNT2_P217_F	0.9880323	2.00E-06	6.00E-06
SOX1_P1018_R	0.787649	2.00E-06	7.00E-06
IGFBP2_P306_F	1.1467117	2.00E-06	7.00E-06

DLL1_P832_F	0.76090352	0.000489	0.001974	CLK1_P538_F	0.6495179	2.00E-06	7.00E-06
CFTR_P372_R	0.9051371	5.00E-04	0.002003	FGF8_P473_F	0.9539725	2.00E-06	8.00E-06
FGFR3_E297_R	0.19122377	0.000513	0.002041	HTR1B_E232_R	0.795426	2.00E-06	8.00E-06
NPR2_P1093_F	-0.2832037	0.000535	0.002111	ALOX12_P223_R	0.82973	2.00E-06	8.00E-06
USP29_P205_R	-0.448621	0.000552	0.002163	APBA2_P305_R	0.6661871	2.00E-06	9.00E-06
GABRA5_E44_R	-0.6233184	0.000557	0.002166	TRPM5_P979_F	0.7726866	2.00E-06	9.00E-06
HOXB2_P488_R	1.04450058	0.000565	0.002183	RBP1_E158_F	0.9021215	3.00E-06	9.00E-06
CSPG2_E38_F	1.07358705	0.00058	0.002214	EPHA2_P340_R	0.7867319	3.00E-06	9.00E-06
S100A2_P1186_F	0.7202364	0.000584	0.002214	MYOD1_E156_F	0.8595843	3.00E-06	9.00E-06
VIM_P811_R	1.35216927	0.000585	0.002214	BCR_P346_F	0.7169858	3.00E-06	1.00E-05
IL1RN_P93_R	0.70117664	0.000599	0.002248	ALOX12_E85_R	0.8112748	3.00E-06	1.00E-05
BMP4_P199_R	0.7343184	0.000605	0.00225	FES_E34_R	0.9875445	3.00E-06	1.10E-05
LAT_E46_F	-0.5188555	0.000607	0.00225	EPHA5_P66_F	0.8177695	3.00E-06	1.10E-05
IL12A_E287_R	0.24358445	0.000612	0.00225	NRG1_E74_F	0.9210729	3.00E-06	1.10E-05
HFE_E273_R	1.45565497	0.000635	0.002314	NTRK3_P752_F	0.87635	3.00E-06	1.10E-05
AOC3_P890_R	0.72261015	0.000638	0.002314	ERG_E28_F	0.7879743	3.00E-06	1.20E-05
S100A4_E315_F	0.76128213	0.000645	0.002326	PLAT_E158_F	0.6708396	4.00E-06	1.20E-05
SERPINA5_P156_F	0.64737256	0.000669	0.002396	RARA_P176_R	0.7652552	4.00E-06	1.30E-05
CHI3L2_P226_F	-0.6139131	0.00068	0.002419	MMP2_E21_R	0.9231266	4.00E-06	1.30E-05
IGSF4_P454_F	1.09450178	0.000715	0.002528	CCND2_P887_F	1.1258284	4.00E-06	1.30E-05
EVI2A_P94_R	-1.0644786	0.000721	0.002532	MYH11_P236_R	1.0876926	4.00E-06	1.40E-05
TJP2_P330_R	-0.7683128	0.00077	0.002686	NPY_P91_F	0.844781	4.00E-06	1.40E-05
SPI1_P48_F	0.74159052	0.000776	0.002689	ETV1_P515_F	0.8433644	4.00E-06	1.40E-05
KRT5_E196_R	0.94545585	0.000803	0.002729	GUCY2D_E419_R	1.0334379	5.00E-06	1.50E-05
SYK_E372_F	0.26943955	0.000803	0.002729	GSTM2_P109_R	0.8845424	5.00E-06	1.50E-05
MFAP4_P10_R	0.53055178	0.000803	0.002729	WNT10B_P823_R	0.7130354	5.00E-06	1.60E-05
GLI2_P295_F	0.57025546	0.000808	0.002729	CCND2_P898_R	1.0730452	5.00E-06	1.60E-05
LIG3_P622_R	0.61853773	0.000818	0.002747	MDR1_seq_42_S30(	1.0270933	5.00E-06	1.80E-05
FASTK_P598_R	0.50930476	0.000849	0.002834	GAS7_E148_F	1.1947071	6.00E-06	1.90E-05
FLJ20712_P984_R	0.63118067	0.000876	0.002877	ADCYAP1_P398_F	0.8909913	6.00E-06	2.00E-05
TAL1_E122_F	1.08502741	0.00088	0.002877	KRT5_E196_R	0.8120604	7.00E-06	2.10E-05
EPHA2_P340_R	0.62945754	0.000883	0.002877	RASGRF1_E16_F	1.1660972	8.00E-06	2.30E-05
HDAC1_P414_R	0.63744508	0.000883	0.002877	IGFBP7_P297_F	0.7159943	8.00E-06	2.30E-05
CD82_P557_R	0.66736941	0.000898	0.002898	SLC14A1_E295_F	0.6587618	8.00E-06	2.50E-05

TRIP6_P1090_F	0.78811179	0.000901	0.002898
EYA4_P794_F	0.9637953	0.000912	0.002918
PTK6_E50_F	0.57246095	0.000943	0.003
APBA2_P305_R	0.62073764	0.000975	0.003083
NOTCH3_P198_R	0.59280305	0.001	0.003143
TGFB2_E226_R	1.00289997	0.001012	0.003163
SH3BP2_E18_F	0.648192	0.001021	0.003163
ZIM3_E203_F	-0.7058678	0.001024	0.003163
FANCA_P1006_R	-0.4931767	0.001071	0.003289
ABCG2_P178_R	0.23043931	0.001103	0.003368
MMP9_P189_F	0.54293027	0.001125	0.003416
EPHA8_P456_R	0.63821233	0.001161	0.003487
MAS1_P657_R	-0.4507262	0.001166	0.003487
NID1_P714_R	0.67534714	0.001167	0.003487
KCNK4_E3_F	0.66999811	0.001203	0.003574
CARD15_P302_R	0.7664463	0.00121	0.003574
TNFRSF10C_P7_F	1.07955802	0.001222	0.00359
MCM2_P260_F	-0.3678257	0.001257	0.003662
RUNX3_P247_F	-0.6525751	0.00126	0.003662
PADI4_E24_F	0.67582712	0.001363	0.003942
SPP1_E140_R	0.61618076	0.001378	0.003963
ENC1_P484_R	0.13396179	0.001435	0.004106
P2RX7_P119_R	0.54606545	0.001483	0.004221
CD1A_P414_R	-0.5966595	0.00153	0.004331
CREBBP_P712_R	0.62613915	0.00154	0.004337
DBC1_E204_F	0.93943156	0.001559	0.004367
AXL_P223_R	0.75650422	0.00159	0.004403
IRF7_P277_R	1.22822109	0.001594	0.004403
TRIP6_P1274_R	0.64771122	0.001596	0.004403
CALCA_P75_F	0.9951615	0.001622	0.004452
DNAJC15_P65_F	-0.3993815	0.001669	0.004559
GSTM2_P109_R	1.0108162	0.001702	0.004625
B3GALT5_P330_F	-0.2272246	0.001783	0.004821
TNFSF8_E258_R	-0.5661305	0.00183	0.004922

TWIST1_E117_R	0.9901746	8.00E-06	2.50E-05
VIM_P811_R	0.9458475	9.00E-06	2.70E-05
AATK_P519_R	0.7257097	9.00E-06	2.90E-05
GPR116_E328_R	0.6264957	1.00E-05	3.00E-05
ASCL2_P360_F	1.1709985	1.00E-05	3.10E-05
FGF2_P229_F	0.7677583	1.10E-05	3.10E-05
EPO_E244_R	1.0565579	1.10E-05	3.20E-05
NID1_P714_R	0.6676746	1.10E-05	3.30E-05
DIO3_P674_F	1.0138525	1.10E-05	3.30E-05
ACVR1_P983_F	0.7211604	1.10E-05	3.30E-05
TFF2_P557_R	0.6414909	1.10E-05	3.30E-05
DCC_E53_R	0.7676856	1.20E-05	3.60E-05
DDR2_P743_R	0.6580906	1.30E-05	3.60E-05
MMP19_E274_R	0.6355384	1.30E-05	3.70E-05
TP73_P945_F	0.8101387	1.40E-05	4.00E-05
NTRK2_P10_F	0.7707377	1.40E-05	4.10E-05
ISL1_P554_F	0.8144283	1.50E-05	4.10E-05
NGFB_E353_F	0.644374	1.50E-05	4.10E-05
TGFB2_E226_R	0.8635648	1.50E-05	4.20E-05
TWIST1_P44_R	0.7447107	1.50E-05	4.30E-05
ERN1_P809_R	0.754174	1.60E-05	4.40E-05
MFAP4_P10_R	0.5965515	1.60E-05	4.40E-05
HTR1B_P107_F	0.6529325	1.60E-05	4.40E-05
JAK3_P1075_R	0.5603623	1.70E-05	4.60E-05
PADI4_E24_F	0.6565798	1.80E-05	4.80E-05
CFTR_P372_R	0.7917552	1.80E-05	4.90E-05
GSTM2_E153_F	0.8872418	1.90E-05	5.00E-05
FGF12_E61_R	0.6799094	1.90E-05	5.10E-05
ADCYAP1_P455_R	0.8269532	1.90E-05	5.10E-05
ZIM2_E110_F	0.6744559	2.00E-05	5.30E-05
P2RX7_P597_F	0.6337276	2.10E-05	5.40E-05
FHIT_P93_R	0.7279659	2.10E-05	5.40E-05
MFAP4_P197_F	0.6606918	2.20E-05	5.60E-05
CALCA_E174_R	0.8532241	2.30E-05	5.80E-05

GP1BB_P278_R	0.92049092	0.001852	0.004957	ABO_P312_F	0.9024249	2.30E-05	5.80E-05
EPHX1_P22_F	0.7466889	0.001879	0.005004	SLC14A1_P369_R	0.6734647	2.30E-05	5.80E-05
CD34_P780_R	0.52462641	0.001999	0.005271	CSF1R_P73_F	0.5545565	2.40E-05	6.10E-05
FLI1_P620_R	1.14815574	0.002008	0.005271	TMPRSS4_E83_F	0.6192091	2.40E-05	6.10E-05
CDK10_P199_R	0.47634905	0.002008	0.005271	FGF5_E16_F	0.7599622	2.50E-05	6.20E-05
EPHA2_P203_F	0.58782732	0.002061	0.005382	SCGB3A1_E55_R	0.8690148	2.70E-05	6.70E-05
RARB_E114_F	1.10141754	0.00207	0.005382	DBC1_P351_R	0.9107145	2.80E-05	7.00E-05
RET_seq_53_S374_F	1.38443296	0.002145	0.005528	HPN_P823_F	0.6653081	3.00E-05	7.40E-05
FGFR1_E317_F	0.25648897	0.002147	0.005528	PROK2_P390_F	1.1329987	3.10E-05	7.60E-05
NID1_P677_F	0.65208593	0.002322	0.005914	HOXA5_E187_F	0.8089707	3.20E-05	7.80E-05
NTSR1_P318_F	1.03139564	0.002322	0.005914	IGF2AS_E4_F	0.5924502	3.30E-05	8.00E-05
PPARD_P846_F	0.2271664	0.00233	0.005914	CSPG2_E38_F	0.7888026	3.40E-05	8.20E-05
TYRO3_P501_F	0.32805906	0.002348	0.005933	PDGFB_P719_F	0.6406528	3.40E-05	8.20E-05
LIF_E208_F	0.17888516	0.002518	0.006332	KRT5_P308_F	0.5596783	3.50E-05	8.50E-05
OGG1_E400_F	0.81583205	0.002721	0.006793	GDF10_E39_F	0.5135615	3.90E-05	9.20E-05
DDR1_P332_R	0.6715059	0.002726	0.006793	EPHA8_P456_R	0.6650507	3.90E-05	9.20E-05
FZD9_P15_R	0.51786551	0.002813	0.006977	P2RX7_P119_R	0.6004559	3.90E-05	9.30E-05
OPCML_E219_R	0.94329631	0.002874	0.00707	CALCA_P75_F	0.8271203	4.00E-05	9.30E-05
GAS7_P622_R	0.77982016	0.002877	0.00707	HHIP_P307_R	0.6700046	4.00E-05	9.40E-05
LTB4R_P163_F	-0.3057962	0.002994	0.007325	MYOD1_P50_F	0.6758737	4.00E-05	9.40E-05
XRCC2_P1077_F	0.78221462	0.003061	0.007456	CPA4_E20_F	0.5630168	4.10E-05	9.60E-05
IRAK3_P13_F	1.1516425	0.003093	0.007499	SOX17_P287_R	0.7663266	4.70E-05	0.000107
ACVR1C_P363_F	0.73547856	0.003161	0.007603	IRF7_E236_R	0.7695841	4.70E-05	0.000107
DHCR24_P652_R	-0.4997944	0.003164	0.007603	USP29_E274_F	-0.6607093	4.70E-05	0.000108
PADI4_P1158_R	0.55090196	0.003183	0.007615	DLC1_P88_R	-0.6686356	4.80E-05	0.00011
NTSR1_E109_F	1.1576792	0.003202	0.007627	GFI1_P45_R	0.887196	4.90E-05	0.000111
SLC5A8_E60_R	0.7603832	0.003305	0.007838	FGF12_P210_R	0.9078274	4.90E-05	0.000111
RASSF1_E116_F	1.40578964	0.003345	0.007897	AHR_E103_F	0.5492885	5.00E-05	0.000113
IGSF4C_P533_R	0.3077147	0.003408	0.007973	FLT1_E444_F	1.1216883	5.10E-05	0.000113
IL3_P556_F	-0.3033045	0.003418	0.007973	S100A2_E36_R	0.5148978	5.10E-05	0.000114
THBS1_E207_R	0.24805874	0.003421	0.007973	PLXDC2_E337_F	1.1854688	5.20E-05	0.000116
SMO_E57_F	1.45496856	0.003478	0.00804	IFNGR2_E164_F	0.399393	5.30E-05	0.000118
PTPRH_P255_F	0.68184654	0.00348	0.00804	MMP9_P189_F	0.5139227	5.40E-05	0.000118
SEMA3B_P110_R	0.36920699	0.003504	0.00804	RBP1_P150_F	0.912626	6.00E-05	0.000132

DST_E31_F	0.28736906	0.003509	0.00804	HOXA5_P1324_F	0.594197	6.10E-05	0.000134
NPR2_P618_F	-0.5740183	0.003548	0.008094	TBX1_P885_R	0.8270241	6.50E-05	0.000142
SRC_P297_F	0.54616337	0.003612	0.008206	PENK_P447_R	0.7367891	6.60E-05	0.000144
RIPK4_E166_F	0.40862421	0.003629	0.00821	UGT1A1_P315_R	0.578955	6.90E-05	0.00015
SOX1_P294_F	0.859011	0.003691	0.008316	COMT_E401_F	0.6777128	7.00E-05	0.000151
FGF3_P171_R	0.94903461	0.003789	0.008501	CD34_P780_R	0.4969116	7.80E-05	0.000167
GUCY2D_P48_R	0.60380378	0.003852	0.008606	PALM2-AKAP2_P42(	0.7224622	7.90E-05	0.000168
ALOX12_P223_R	0.73940042	0.003947	0.008781	GJB2_P791_R	0.9737766	8.00E-05	0.000169
CASP10_P186_F	0.41291194	0.003998	0.008859	KDR_P445_R	0.8841513	8.10E-05	0.000171
HS3ST2_E145_R	0.80375356	0.004033	0.0089	KCNK4_P171_R	0.554081	8.10E-05	0.000171
HRASLS_P353_R	0.50405425	0.004141	0.009101	TMPRSS4_P552_F	0.7119793	8.50E-05	0.000177
ITGB4_E144_F	0.1532299	0.00417	0.009128	TUBB3_E91_F	0.5908033	8.80E-05	0.000184
FGF3_E198_R	1.07798922	0.004212	0.009183	PLA2G2A_P528_F	0.5042009	8.80E-05	0.000184
TUBB3_E91_F	0.69924861	0.004288	0.009297	TNF_P158_F	0.6592584	8.90E-05	0.000185
NRG1_E74_F	0.90285302	0.004325	0.009297	PDGFRB_P273_F	0.555132	9.10E-05	0.000187
SNCG_P98_R	0.44988888	0.004329	0.009297	MEG3_E91_F	0.5992403	9.10E-05	0.000187
ZIM2_E110_F	0.40018258	0.004333	0.009297	GLI2_P295_F	0.5978083	9.40E-05	0.000193
JAK3_E64_F	0.98380059	0.004648	0.009932	POMC_E254_F	0.6364024	9.50E-05	0.000194
TNFRSF10C_E109_F	0.92008397	0.004696	0.009937	FLT3_P302_F	0.9801679	9.70E-05	0.000198
DES_P1006_R	0.4266252	0.004698	0.009937	IL18BP_P51_R	0.5135745	0.000105	0.000213
FGF2_P229_F	0.80337089	0.004705	0.009937	RARB_E114_F	0.8364273	0.000107	0.000215
CAPG_E228_F	0.42120249	0.00487	0.01021	SLC5A8_P38_R	0.7712563	0.000112	0.000226
MYCN_E77_R	0.20809526	0.004885	0.01021	IGFBP2_P353_R	0.6084737	0.000117	0.000234
NOTCH1_P1198_F	0.50104906	0.004895	0.01021	TNFRSF1A_P678_F	0.5260996	0.000124	0.000246
EVI2A_E420_F	-0.4377537	0.00491	0.01021	PTPRO_E56_F	1.038609	0.000124	0.000246
HLA-DOA_P191_R	0.44447431	0.005032	0.010388	PYCARD_P393_F	0.5198856	0.00013	0.000256
GALR1_E52_F	0.88082491	0.005034	0.010388	TRIM29_P261_F	0.591038	0.000131	0.000258
VIM_P343_R	1.02044243	0.005107	0.0105	SEZ6L_P249_F	0.8570432	0.000134	0.000263
TNFRSF1A_P678_F	0.53245963	0.00523	0.010712	PLAT_P80_F	0.6906381	0.000141	0.000275
TPEF_seq_44_S88_R	0.83719366	0.00531	0.010833	ADAMTS12_E52_R	0.6846306	0.000141	0.000275
NEU1_P745_F	-0.7794576	0.005491	0.01114	CSF2_E248_R	0.5900853	0.000144	0.00028
ETV6_E430_F	0.28233184	0.005501	0.01114	IRF7_P277_R	0.9509626	0.000155	0.000299
POMC_P53_F	1.25796147	0.005539	0.011174	GUCY2D_P48_R	0.5252958	0.00017	0.000327
CDH13_E102_F	0.9826633	0.005561	0.011177	NBL1_P24_F	0.5995071	0.000178	0.00034

SLIT2_E111_R	0.72907708	0.005597	0.011207	HLA-F_E402_F	1.0150306	0.00018	0.000343
MST1R_E42_R	0.76819841	0.005629	0.011231	ALK_E183_R	0.5249569	0.00018	0.000343
MLH3_P25_F	0.28961982	0.005687	0.011279	NEFL_P209_R	0.6877296	0.000182	0.000345
WNT10B_P823_R	0.64344214	0.005697	0.011279	ADCYAP1_E163_R	0.7868616	0.000185	0.00035
HIC2_P498_F	0.651182	0.005716	0.011279	TRIP6_P1090_F	0.5651035	0.000192	0.000361
SHH_E328_F	0.47709709	0.005914	0.011628	SLC22A3_E122_R	0.6595209	0.000206	0.000387
IGF2AS_E4_F	0.48291726	0.00609	0.01193	MAPK10_E26_F	0.657253	0.000207	0.000388
ASCL2_E76_R	0.52997952	0.006115	0.011936	ST6GAL1_P164_R	0.7143181	0.000215	4.00E-04
IGFBP3_P1035_F	-1.2250081	0.006172	0.012004	PTPRO_P371_F	0.6324508	0.000216	4.00E-04
CASP10_P334_F	0.45748392	0.006233	0.012018	EPHA7_E6_F	0.8709245	0.000223	0.000412
ERG_E28_F	0.70747912	0.006241	0.012018	KCNK4_E3_F	0.5420987	0.000224	0.000412
POMC_P400_R	0.97457377	0.006251	0.012018	ABO_E110_F	0.7631019	0.000225	0.000412
SFTPC_E13_F	0.42940444	0.006268	0.012018	TYRO3_P501_F	0.3039389	0.000225	0.000412
SPI1_P929_F	0.37316841	0.006441	0.012305	APC_P14_F	0.6844019	0.000231	0.000421
MET_E333_F	0.71290781	0.006491	0.012358	CSF1_P339_F	0.7236148	0.000241	0.000437
LMO1_E265_R	0.63111841	0.00661	0.01254	HLF_E192_F	0.7394887	0.000242	0.000438
CCND1_P343_R	0.45285181	0.006714	0.012693	PDE1B_E141_F	0.5469152	0.000254	0.000458
CTGF_E156_F	0.50523076	0.006824	0.012855	SMO_P455_R	0.7128308	0.000261	0.000469
THY1_P149_R	0.76491692	0.007039	0.013197	SEMA3C_P642_F	-0.7224159	0.000263	0.000471
CCL3_E53_R	-0.521407	0.007054	0.013197	MATK_P64_F	0.6682893	0.000266	0.000475
TFF2_P557_R	0.52333256	0.007133	0.013298	DAB2IP_E18_R	0.5906588	0.000286	0.000508
COL1A2_P48_R	0.98429427	0.007212	0.013319	FLT1_P302_F	0.7436849	0.000292	0.000517
TMPRSS4_P552_F	0.58625542	0.007217	0.013319	MYH11_P22_F	0.7172948	0.000296	0.000523
CALCA_E174_R	0.79585957	0.007218	0.013319	SYK_P584_F	0.4771882	0.000297	0.000523
ABCC5_P444_F	0.30061212	0.00728	0.013388	SFTPA1_E340_R	0.6252805	0.000309	0.00054
MST1R_P87_R	0.6859889	0.007334	0.013418	MMP9_P237_R	0.6467792	0.000309	0.00054
FRZB_P406_F	1.13324092	0.007346	0.013418	AXL_P223_R	0.5777484	0.000322	0.00056
TK1_P62_R	0.10679644	0.007378	0.013432	DHCR24_P652_R	-0.4528766	0.000326	0.000566
AFF3_P808_F	-0.5003423	0.007428	0.013477	GABRG3_P75_F	-0.5616587	0.00033	0.000572
PLXDC1_P236_F	0.49255279	0.00753	0.013616	AATK_P709_R	0.6209984	0.000335	0.000577
NPY_E31_R	0.7107049	0.00756	0.013625	KLK10_P268_R	0.4981984	0.000337	0.000579
DDR2_P743_R	0.55662381	0.007795	0.014003	IL1RN_P93_R	0.5459886	0.000367	0.000629
CHD2_P667_F	-0.4101142	0.007831	0.014008	TMEFF1_P626_R	0.4418576	0.000371	0.000633
ERCC6_P698_R	0.31359961	0.00785	0.014008	PTHR1_P258_F	0.4491882	0.000385	0.000656

TP73_P496_F	0.81083639	0.007926	0.014084	NTRK3_E131_F	0.8831409	0.000393	0.000667
NPY_P295_F	0.79826623	0.007945	0.014084	EPHA2_P203_F	0.5333832	0.000398	0.000673
GSTM2_E153_F	0.87171173	0.008072	0.014263	FAS_P322_R	0.5285867	0.00041	0.00069
CTSD_P726_F	0.36924898	0.008135	0.014289	PDGFRB_E195_R	0.7898113	0.000421	0.000707
MC2R_P1025_F	-0.461997	0.00814	0.014289	CSF1R_E26_F	0.5632405	0.000445	0.000744
GRB10_E85_R	0.65149344	0.008269	0.014468	MATK_P190_R	0.5939265	0.000453	0.000756
MMP19_P306_F	0.46580318	0.008308	0.01449	FLT4_P180_R	0.8721951	0.000459	0.000763
ERBB4_P541_F	0.29806081	0.008526	0.014782	DAB2IP_P9_F	0.6349309	0.000462	0.000765
HLA-DQA2_P282_R	-0.4663334	0.00853	0.014782	FGFR3_E297_R	0.3493222	0.000463	0.000765
EPHB2_E297_F	0.15891797	0.00868	0.014971	OAT_P465_F	0.600431	0.000489	0.000805
PPARG_E178_R	0.13794581	0.00872	0.014971	TAL1_P594_F	0.6308686	0.000497	0.000816
DAPK1_P345_R	0.39117574	0.008722	0.014971	FGF5_P238_R	0.6629123	0.000509	0.000834
ITK_E166_R	-0.5833358	0.009097	0.015564	COL1A2_E299_F	0.5890652	0.000513	0.000835
HIC-1_seq_48_S103_	-0.8337582	0.009187	0.015648	CYP1B1_E83_R	0.7300577	0.000514	0.000835
RIPK3_P124_F	0.46595413	0.009219	0.015648	SOX17_P303_F	0.5356114	0.000515	0.000835
DNMT3B_P352_R	0.45836441	0.009232	0.015648	TFPI2_P9_F	0.8481601	0.000526	0.00085
PYCARD_P150_F	0.39925852	0.009323	0.015746	ZIM2_P22_F	0.5572286	0.000531	0.000854
CEACAM1_E57_R	0.27188402	0.009348	0.015746	NGFB_P13_F	0.5542879	0.000532	0.000854
BMPR2_E435_F	-0.1530988	0.009388	0.015763	ALPL_P433_F	0.455303	0.000563	0.000901
TMEFF2_P152_R	0.67235909	0.009456	0.015828	DNAJC15_E26_R	0.4962734	0.000565	0.000903
TJP2_P518_F	-0.6306357	0.009504	0.015859	PTPRG_E40_R	0.6615916	0.000576	0.000917
IGF1R_E186_R	0.14270745	0.009681	0.016105	SPARC_E50_R	0.4915722	6.00E-04	0.000953
GSTM2_P453_R	0.52092759	0.010076	0.016712	RARB_P60_F	0.5688924	0.000606	0.000959
CRK_P721_F	0.37618545	0.010547	0.017439	TGFB1_P833_R	0.6845644	0.000632	0.000997
SEZ6L_P249_F	1.25649971	0.010599	0.017471	ER_seq_a1_S60_F	0.5386019	0.000647	0.001017
SEZ6L_P299_F	1.02646791	0.01072	0.017584	ESR1_E298_R	0.5918152	0.000649	0.001018
EPO_P162_R	0.56666778	0.010732	0.017584	PAX6_P1121_F	0.5831069	0.000672	0.00105
TP73_P945_F	0.69805308	0.010906	0.017732	GJB2_P931_R	0.5630656	0.000694	0.001078
MYCN_P464_R	0.1527002	0.010912	0.017732	BMP6_P163_F	0.4077947	0.000694	0.001078
ZMYND10_E77_R	0.81888673	0.010921	0.017732	THBS2_P605_R	0.4599146	0.000714	0.001106
SPDEF_P6_R	0.47095989	0.011096	0.017963	SNCG_E119_F	0.6030105	0.000743	0.001146
IL1B_P582_R	0.41900956	0.011184	0.018051	FGF8_E183_F	0.4517903	0.000784	0.001207
TGFA_P642_R	0.29477815	0.01128	0.018152	ERBB4_P541_F	0.7142247	0.000789	0.001211
AGTR1_P154_F	0.97186072	0.011348	0.018206	TRPM5_P721_F	0.505481	0.00083	0.001269

ZIM3_P718_R	-0.5183679	0.011472	0.01835	CRIP1_P874_R	0.5454827	0.000832	0.001269
EPHA1_P119_R	0.33588728	0.011569	0.018451	ST6GAL1_P528_F	0.6598448	0.000843	0.001282
MMP3_P16_R	-0.629172	0.011975	0.018993	HOXA5_P479_F	0.5824461	0.000949	0.001439
MKRN3_P108_F	-0.5065892	0.012043	0.018993	DLC1_E276_F	-0.5575026	0.000965	0.00146
IGFBP2_P353_R	0.6477938	0.012048	0.018993	CASP6_P230_R	0.1313071	0.000981	0.001479
IGFBP6_E47_F	0.24677198	0.012049	0.018993	PLAU_P176_R	0.626141	0.001004	0.001509
OAT_P465_F	0.78838566	0.01212	0.01905	GRB10_P496_R	0.4948693	0.001013	0.001519
SNRPN_P230_R	-0.2939649	0.012181	0.019089	GABRB3_E42_F	0.7122841	0.001039	0.001554
IRAK3_P185_F	0.85705461	0.012261	0.019159	ADAMTS12_P250_R	0.7972399	0.001045	0.001555
KCNQ1_P546_R	0.32376024	0.012354	0.019249	CDK6_P291_R	0.7309284	0.001046	0.001555
ZNFN1A1_P179_F	-0.6072922	0.012828	0.01993	F2R_P88_F	0.6717445	0.001067	0.001581
PHLDA2_P622_F	0.40331082	0.012907	0.019996	GP1BB_E23_F	0.7716425	0.001108	0.001637
RASSF1_P244_F	1.16835288	0.013027	0.020091	SERPINE1_P519_F	0.5140538	0.001171	0.001725
EDN1_E50_R	0.26075072	0.013057	0.020091	IGFBP7_P371_F	0.7350786	0.00123	0.001806
SEMA3A_P658_R	0.39775221	0.01312	0.020091	TSP50_P137_F	0.5771877	0.001233	0.001806
MYOD1_E156_F	0.7407076	0.013135	0.020091	CCKBR_P480_F	0.6883218	0.001238	0.001809
WNT1_E157_F	0.51297163	0.013155	0.020091	TIMP3_seq_7_S38_I	0.7883103	0.001286	0.001873
EFNB3_P442_R	0.23770479	0.013228	0.020146	RET_seq_53_S374_F	0.685152	0.00129	0.001874
GLI3_E148_R	-0.2917399	0.013362	0.020294	CDKN1C_P626_F	0.7203593	0.001368	0.001982
PROK2_E0_F	0.33825232	0.013429	0.020338	S100A4_P194_R	0.5489954	0.001423	0.002056
ERCC1_P440_R	0.25861682	0.01364	0.020601	GPX3_E178_F	0.4830923	0.001445	0.002081
SLC22A18_P472_R	-0.352946	0.013803	0.020789	BDNF_E19_R	0.536973	0.001477	0.002121
BCAM_P205_F	0.24119023	0.013845	0.020793	NID1_P677_F	0.4812568	0.001484	0.002126
MGMT_P281_F	0.21020884	0.013994	0.020959	ALK_P28_F	0.580235	0.001564	0.002235
TSP50_P137_F	0.72431981	0.01416	0.021149	TIMP2_P267_F	0.6233039	0.001581	0.002252
S100A2_E36_R	0.33825045	0.014233	0.0212	MKRN3_E144_F	-0.6287496	0.001654	0.00235
HIC2_P528_R	0.59539748	0.014667	0.021786	KIT_P405_F	0.6393987	0.001667	0.002356
MATK_P64_F	0.61866766	0.014749	0.021848	SERPINA5_P156_F	0.4712239	0.001667	0.002356
IGFBP3_P423_R	-0.8948478	0.015111	0.022323	PTPNS1_P301_R	0.2629415	0.001675	0.00236
EPO_E244_R	0.91563506	0.015167	0.022344	PSCA_E359_F	0.3811293	0.0017	0.002389
ZAP70_P220_R	0.36631769	0.015505	0.022781	FGFR1_E317_F	0.3659824	0.001829	0.002562
CTNNA1_P382_R	0.16083147	0.015835	0.023202	SEMA3B_E96_F	0.4078033	0.001857	0.002595
GLI3_P453_R	0.66963773	0.015961	0.023324	PTPRH_E173_F	0.3899539	0.001905	0.002655
NPY_P91_F	0.73374788	0.016488	0.023972	PYCARD_E87_F	0.7033255	0.00213	0.002961

WNT2_P217_F	0.72720518	0.016494	0.023972
IRAK3_E130_F	0.93680135	0.01675	0.024232
EVI1_E47_R	0.33806	0.016762	0.024232
TNFRSF10D_P70_F	0.8064833	0.016837	0.024276
RBL2_P250_R	0.64541989	0.017001	0.024414
RARRES1_E235_F	0.57412782	0.017066	0.024414
KCNQ1_E349_R	0.19195826	0.017069	0.024414
TNFRSF10D_E27_F	0.87918201	0.017209	0.02455
TERT_E20_F	0.55384907	0.017478	0.024867
CCKBR_P480_F	0.88372554	0.01756	0.024919
EIF2AK2_P313_F	0.7538059	0.017937	0.025388
PWCR1_P811_F	-0.3419782	0.018089	0.025536
MMP2_P197_F	0.59730414	0.018265	0.025718
NR2F6_E375_R	0.21962602	0.018646	0.026125
PROK2_P390_F	1.04495961	0.018651	0.026125
LMO2_E148_F	-0.3735794	0.018754	0.026201
PWCR1_E81_R	-0.3540959	0.018978	0.026445
SLC5A8_P38_R	0.69402669	0.01934	0.026881
PKD2_P287_R	0.21941445	0.019444	0.026956
NCL_P840_R	0.32481815	0.019571	0.027063
SEMA3C_P642_F	-0.6871592	0.019762	0.027257
HLA-DPA1_P205_R	0.39375623	0.019914	0.027397
PLA2G2A_P528_F	0.39429937	0.020022	0.027476
ADCYAP1_P455_R	0.73806319	0.020101	0.027514
FLT3_P302_F	0.9513125	0.020613	0.028143
MFAP4_P197_F	0.46164146	0.021039	0.028585
GRB7_P160_R	0.44014802	0.021042	0.028585
SPP1_P647_F	0.42200197	0.021458	0.029077
HIC1_P565_R	0.36278542	0.021589	0.029181
BAX_E281_R	0.28407404	0.021791	0.029381
RBP1_E158_F	0.86217483	0.021932	0.029497
FES_E34_R	0.5862086	0.022293	0.029908
THPO_E483_F	0.5331291	0.022367	0.029933
MCM2_P241_R	0.12074135	0.022646	0.030233

SEZ6L_P299_F	0.6925701	0.002141	0.002967
PLXDC1_P236_F	0.5880126	0.002195	0.003033
KDR_E79_F	0.724335	0.002203	0.003033
CCNA1_E7_F	0.6131215	0.002205	0.003033
GABRB3_P92_F	0.3688487	0.00225	0.003086
TDGF1_E53_R	0.4513659	0.002303	0.003151
PDGFRA_P1429_F	0.5598464	0.002313	0.003156
TNFRSF10D_E27_F	0.6659908	0.002323	0.00316
CD40_E58_R	0.6093257	0.002373	0.003216
ERCC1_P354_F	0.3616341	0.002376	0.003216
LOX_P313_R	0.5155334	0.002384	0.003218
PTHLH_P757_F	0.4714468	0.002587	0.003483
PTHLH_E251_F	0.4248722	0.002617	0.003515
ASCL1_E24_F	0.6788192	0.002661	0.003565
ESR2_P162_F	0.4697933	0.00268	0.003581
ETV6_E430_F	0.3434337	0.002703	0.003602
AIM2_P624_F	0.4685859	0.00274	0.003642
MAPK12_P416_F	0.646521	0.002839	0.003764
TJP1_P390_F	0.2585284	0.002896	0.00383
TCF4_P175_R	0.6115738	0.002905	0.003831
TGFB2_P632_F	0.5256692	0.002937	0.003864
HHIP_P578_R	0.4177633	0.003045	0.003996
FN1_E469_F	0.5573874	0.003075	0.004025
NKX3-1_P871_R	0.5925441	0.003119	0.004073
RIPK3_P124_F	0.4496086	0.003205	0.004174
GLI3_P453_R	0.4896281	0.003248	0.004219
GSTP1_E322_R	0.5461881	0.003389	0.004392
CFTR_P115_F	0.6312841	0.003481	0.004499
IGF2AS_P203_F	0.5351591	0.003498	0.00451
MMP7_E59_F	0.3528744	0.003553	0.004569
BDNF_P259_R	0.4885245	0.003576	0.004587
SIN3B_P607_F	-0.5428856	0.00372	0.004761
COL18A1_P494_R	0.5429194	0.00378	0.004826
SIN3B_P514_R	-0.4888275	0.0038	0.004839

PDGFA_P841_R	0.12627188	0.022757	0.030302
ERBB4_P255_F	0.17746559	0.022811	0.030302
GP1BB_E23_F	0.94965609	0.022968	0.030436
H19_P1411_R	-0.4431781	0.023139	0.030588
ICA1_P72_R	0.21018428	0.023645	0.031181
RIPK3_P24_F	0.59907158	0.023783	0.031287
SLC22A3_E122_R	0.74756517	0.023878	0.031335
LRRK1_P39_F	-0.1528137	0.024169	0.031588
OPCML_P71_F	0.46697588	0.024187	0.031588
RARRES1_P426_R	0.50319659	0.024386	0.031699
CDC25B_E83_F	1.14626367	0.02439	0.031699
DIO3_P674_F	0.82781334	0.024504	0.031772
IGF1_E394_F	0.50976015	0.024884	0.032187
COL1A1_P5_F	0.38682956	0.025346	0.032705
LIF_P383_R	0.41172807	0.025406	0.032705
TMEFF2_P210_R	0.50524877	0.025546	0.032808
ERBB3_E331_F	0.30405395	0.026136	0.033486
HBII-13_P991_R	-0.2150952	0.026361	0.033621
GDF10_E39_F	0.31938086	0.026366	0.033621
APC_E117_R	0.65701807	0.026452	0.033651
CRIP1_P874_R	0.5413196	0.026665	0.033843
PPARG_P693_F	0.47225357	0.027004	0.034192
CEBPA_P1163_R	0.71430656	0.027096	0.034229
THY1_P20_R	0.42441286	0.027397	0.034528
IHH_E186_F	0.77122904	0.027529	0.034537
EYA4_P508_F	0.64342081	0.027532	0.034537
MAD2L1_E93_F	-0.3478083	0.027652	0.034608
ESR1_P151_R	-0.5993691	0.027742	0.03464
SMO_P455_R	0.69410682	0.028217	0.035152
HTR1B_P222_F	0.8359198	0.028532	0.035463
MYH11_P236_R	0.8988397	0.028843	0.035768
CD9_P504_F	0.48371033	0.029576	0.036593
BMP6_P163_F	0.4640087	0.029756	0.036731
MT1A_P49_R	1.07775652	0.03012	0.037096

EPO_P162_R	0.4518966	0.0041	0.005208
IL6_P213_R	0.5685041	0.00411	0.005208
SPI1_P48_F	0.4777118	0.004143	0.005237
PAX6_E129_F	0.542147	0.004195	0.005288
MEG3_P235_F	0.5210771	0.004204	0.005288
PAX6_P50_R	0.7389189	0.004227	0.005304
CCKBR_P361_R	0.348613	0.004309	0.005394
LCN2_P141_R	0.430597	0.004335	0.005413
HCK_P858_F	0.5270761	0.004364	0.005432
PLA2G2A_E268_F	0.4204733	0.004379	0.005432
HLA-DOB_E432_R	0.43226	0.004382	0.005432
COL1A1_P5_F	0.4262516	0.004482	0.005543
DAPK1_E46_R	0.4766793	0.004527	0.005585
SLC22A3_P634_F	0.4360788	0.00464	0.005711
GABRA5_E44_R	-0.4102028	0.004665	0.005728
EPHA8_P256_F	0.4178603	0.004698	0.005755
PTCH2_P37_F	0.4664633	0.004735	0.005787
SEMA3A_P658_R	0.4056909	0.004786	0.005835
RARA_E128_R	0.5828721	0.004928	0.005994
AKT1_P310_R	0.4348157	0.00508	0.006165
EPHX1_P22_F	0.5277549	0.005127	0.006206
SLC22A3_P528_F	0.4755691	0.00518	0.006256
IGFBP6_E47_F	0.3061653	0.005329	0.006421
TIAM1_P188_R	0.7704165	0.005387	0.006476
RAP1A_P285_R	0.3177505	0.005402	0.006479
DLL1_P832_F	0.4449693	0.00549	0.006568
IGFBP6_P328_R	0.4608132	0.005651	0.006746
IGSF4_P86_R	0.5036669	0.00572	0.006813
PTPN6_P282_R	0.404404	0.00584	0.006939
HRASLS_P353_R	0.4246221	0.005883	0.006974
PGR_P456_R	-0.4779416	0.005954	0.007043
POMC_P53_F	0.6894667	0.006015	0.007098
DST_E31_F	0.1945454	0.006063	0.007139
MT1A_P600_F	0.4475663	0.006136	0.007208

WT1_E32_F	0.76527128	0.030345	0.037288	CALCA_P171_F	0.4087652	0.006216	0.007286
NEFL_E23_R	0.56109835	0.030443	0.037307	MOS_P746_F	-0.4297909	0.006544	0.007653
PITX2_E24_R	-0.6422004	0.030499	0.037307	FGFR3_P1152_R	0.4411221	0.006586	0.007684
CDKN1C_P6_R	0.26009792	0.030577	0.037318	NTRK3_P636_R	0.4531697	0.006624	0.007707
GFI1_P208_R	0.50789292	0.03071	0.037397	APC_P280_R	0.6754614	0.006641	0.007707
UGT1A7_P751_R	-0.2113974	0.030838	0.037467	FGFR1_P204_F	0.5193956	0.00665	0.007707
CDH11_P203_R	0.78319227	0.030907	0.037468	APBA2_P227_F	0.5083915	0.006719	0.007769
SFRP1_E398_R	0.86557448	0.031601	0.038223	AIM2_E208_F	0.3511164	0.006779	0.007821
HHIP_E94_F	0.68068726	0.032125	0.03877	ITK_E166_R	-0.5147863	0.006934	0.007982
KRAS_P651_F	0.1512373	0.032252	0.038777	WNT5A_E43_F	0.3557902	0.006957	0.00799
APBA2_P227_F	0.51651164	0.032335	0.038777	TMEFF2_P210_R	0.4656872	0.0072	0.008252
IL2_P607_R	0.42456446	0.032412	0.038777	ATP10A_P524_R	-0.4496384	0.007285	0.008314
IGF2AS_P203_F	0.55478187	0.032418	0.038777	DDR1_P332_R	0.4454953	0.007298	0.008314
TWIST1_P44_R	0.56512387	0.032571	0.038866	NGFR_P355_F	0.3300846	0.007303	0.008314
PXN_P308_F	0.37324579	0.032688	0.038866	FLT3_E326_R	0.4396557	0.007345	0.008344
SOX1_P1018_R	0.52710264	0.032708	0.038866	HIC1_P565_R	0.4315953	0.007472	0.008469
MMP14_P208_R	0.40047586	0.033065	0.039204	DAB2_P468_F	0.6019166	0.007537	0.008519
SOX17_P303_F	0.57529513	0.033301	0.039398	CCL3_E53_R	-0.3508093	0.007549	0.008519
PTPRH_E173_F	0.29577549	0.03371	0.039795	CDKN1A_E101_F	0.2306949	0.007647	0.008609
WNT10B_P993_F	0.57109105	0.033831	0.039812	RET_seq_54_S260_F	0.6763495	0.007662	0.008609
APC_P14_F	0.71833906	0.033872	0.039812	TFF1_P180_R	0.3748634	0.007776	0.008717
FGFR3_P1152_R	0.55521388	0.034258	0.040179	CTSH_E157_R	0.379037	0.007798	0.008724
MATK_P190_R	0.5136038	0.035204	0.041199	PODXL_P1341_R	0.5224768	0.008171	0.009121
PTCH_E42_F	0.10480772	0.035801	0.041769	NOS2A_E117_R	0.3735451	0.008444	0.009405
TMEFF2_E94_R	0.55169279	0.035846	0.041769	HBII-52_E142_F	-0.3829088	0.008909	0.009902
F2R_P88_F	0.58879443	0.036384	0.042305	ALPL_P278_F	0.617185	0.009035	0.01002
FGF7_P44_F	0.37395204	0.036622	0.04249	GSTM2_P453_R	0.4229832	0.009119	0.010091
IGF2R_P396_R	0.18275254	0.036818	0.042626	TWIST1_P355_R	0.4199551	0.009166	0.010122
IGFBP2_P306_F	0.876915	0.036939	0.042627	ASCL1_P747_F	0.4356448	0.009228	0.010169
EGF_E339_F	-0.496812	0.036976	0.042627	NTRK1_E74_F	-0.5689166	0.009314	0.010242
PLAGL1_E68_R	-0.3057468	0.03719	0.042782	SRC_P164_F	0.3351912	0.00965	0.010582
TNFRSF10A_P91_F	0.29136669	0.037318	0.042838	IGF1R_P325_R	0.2500777	0.009682	0.010582
VAV1_E9_F	0.64926406	0.038001	0.043529	EVI1_E47_R	0.6142481	0.009701	0.010582
CD9_P585_R	0.56414631	0.038414	0.043878	KCNQ1_P546_R	0.3116799	0.009706	0.010582

WRN_P969_F	0.57633568	0.038488	0.043878
WNT5A_P655_F	-0.3553592	0.038548	0.043878
MMP9_E88_R	0.51440928	0.038723	0.043944
TM7SF3_P1068_R	-0.735334	0.038769	0.043944
CDC25B_P11_R	0.7772398	0.039064	0.044186
CTLA4_E176_R	-0.3755866	0.039978	0.045072
CFTR_P115_F	0.69066535	0.040014	0.045072
EXT1_E197_F	0.23926248	0.040223	0.045199
SNRPN_seq_18_S99_	-0.2529063	0.040294	0.045199
GABRB3_P92_F	0.46479986	0.041102	0.046009
ASCL2_P360_F	0.5841272	0.041234	0.046062
PLXDC2_E337_F	0.95342681	0.041511	0.046137
PDGFB_P719_F	0.45565587	0.041514	0.046137
ADAMTS12_P250_R	1.19786986	0.041557	0.046137
HTR1B_E232_R	0.57572073	0.041957	0.046486
TNK1_P41_R	0.42115343	0.042059	0.046503
COL1A2_E299_F	0.59737171	0.042566	0.046845
ONECUT2_P315_R	0.86674268	0.042612	0.046845
MEG3_E91_F	0.34391921	0.042628	0.046845
MAPK12_E165_R	0.19966026	0.043194	0.047325
MT1A_E13_R	0.60667378	0.043243	0.047325
TMEFF1_P626_R	0.27996047	0.043328	0.047325
HCK_P858_F	0.51855311	0.043582	0.047507
LCN2_P86_R	0.44308965	0.043815	0.047609
DES_E228_R	0.55982083	0.043851	0.047609
TIE1_E66_R	-0.4210586	0.044149	0.047836
PITX2_P183_R	-0.4180182	0.044283	0.047885
FAT_P279_R	0.43804593	0.044548	0.048075
AKT1_P310_R	0.17554382	0.044891	0.048349
GNG7_E310_R	-0.2612376	0.045222	0.048609
CARD15_P665_F	-0.4084178	0.045446	0.048753
GRB7_E71_R	0.4281113	0.045752	0.048984
RARB_P60_F	0.39998266	0.046354	0.049347
CEBPA_P706_F	-0.2443305	0.046354	0.049347

CHGA_P243_F	0.3368805	0.01015	0.011043
DSP_P440_R	0.3203455	0.010375	0.011264
IL1A_E113_R	0.6614252	0.010474	0.011348
SNURF_E256_R	-0.3781303	0.010502	0.011354
ZMYND10_E77_R	0.6473478	0.010572	0.011406
SERPINB5_P19_R	0.363559	0.0108	0.011627
AOC3_P890_R	0.3688718	0.010938	0.011752
SEPT5_P464_R	0.3935515	0.010967	0.011758
DES_P1006_R	0.3651744	0.011013	0.011783
BMP2_E48_R	0.4973944	0.01106	0.011809
SNCG_P53_F	0.4377498	0.011246	0.011983
SGCE_E149_F	0.5894232	0.011283	0.011997
TFF2_P178_F	0.3589204	0.011496	0.012199
MBD2_P233_F	0.3463645	0.011773	0.012467
ZAP70_P220_R	0.3624863	0.01189	0.012565
COL4A3_E205_R	0.3778287	0.011971	0.012624
TGFB3_E58_R	0.3510331	0.012335	0.012982
TFPI2_P152_R	0.531965	0.012701	0.01334
IL17RB_E164_R	0.554745	0.012728	0.013341
MCAM_P265_R	0.4238801	0.012858	0.01345
MMP19_P306_F	0.347906	0.012896	0.013463
PLXDC1_E71_F	0.4162346	0.013035	0.01358
TSC2_E140_F	0.4684838	0.013406	0.013938
RARRES1_P426_R	0.398091	0.013514	0.014022
PURA_P928_R	0.5086498	0.014275	0.014768
MLH3_P25_F	0.2941078	0.01429	0.014768
TIAM1_P117_F	0.6398701	0.014342	0.014793
MAGEL2_E166_R	-0.4676024	0.015131	0.015575
NDN_P1110_F	-0.4657434	0.015296	0.015714
S100A4_P887_R	0.488409	0.015586	0.01598
APC_E117_R	0.3370216	0.015811	0.016178
MET_E333_F	0.4006372	0.015994	0.01632
HLA-DOA_P191_R	0.3805932	0.016013	0.01632
LCN2_P86_R	0.3717292	0.016247	0.016526

CDH11_E102_R	0.69713138	0.046365	0.049347
BMP3_P56_R	0.13491412	0.046531	0.049426
GFI1_P45_R	0.82134509	0.046749	0.049561
FLT1_P615_R	0.66965579	0.046863	0.049584
EGF_P242_R	0.31600344	0.047075	0.049711
ALPL_P433_F	0.37680436	0.047181	0.049726

PTK6_E50_F	0.3561884	0.016441	0.016691
RIPK3_P24_F	0.4383822	0.016749	0.016962
NOS3_P38_F	0.3231185	0.016775	0.016962
GFI1_E136_F	0.5097959	0.016934	0.01709
IGF2_P36_R	0.5150913	0.017	0.017123
CLDN4_P1120_R	0.3340416	0.017211	0.017302
ITGA6_P718_R	0.5458422	0.017366	0.017378
CD2_P68_F	0.3401496	0.017374	0.017378
TAL1_P817_F	0.4504493	0.017388	0.017378
GRB10_P260_F	0.573898	0.017592	0.017548
WNT1_P79_R	0.3858318	0.017754	0.017659
FANCA_P1006_R	-0.4160755	0.017797	0.017659
CD1A_P6_F	-0.4234271	0.017807	0.017659
APOA1_P261_F	0.4159841	0.019333	0.019136
P2RX7_E323_R	0.372506	0.019514	0.019278
LRP2_E20_F	0.4297795	0.019788	0.019512
NOTCH1_P1198_F	0.4328952	0.020345	0.020023
THPO_E483_F	0.4840984	0.020479	0.020116
MYB_P673_R	0.1485593	0.020619	0.020216
PSCA_P135_F	0.2990044	0.020871	0.020424
RAB32_E314_R	0.4320883	0.021052	0.020532
RET_P717_F	0.3850125	0.021075	0.020532
HDAC9_E38_F	0.4431337	0.021101	0.020532
GSTP1_P74_F	0.335838	0.021269	0.020656
ERBB4_P255_F	0.4642639	0.021378	0.020723
MMP14_P208_R	0.3703327	0.022152	0.021433
CDH17_P376_F	0.3592099	0.022424	0.021645
RIPK1_P744_R	0.480059	0.022455	0.021645
EDNRB_P148_R	-0.3861181	0.023332	0.022448
KIAA0125_E29_F	-0.3657396	0.02359	0.022654
CYP1B1_P212_F	0.344542	0.023764	0.022779
PKD2_P336_R	0.2443228	0.023998	0.02296
LMO1_E265_R	0.3961843	0.02422	0.02313
HIC2_P498_F	0.5303314	0.024392	0.023252

TRIP6_P1274_R	0.3542444	0.024487	0.023299
ACTG2_E98_R	0.3754015	0.024735	0.023491
IL1RN_E42_F	0.3102412	0.025149	0.02383
EDNRB_P709_R	-0.3639166	0.025184	0.02383
FAS_P65_F	0.3043792	0.025349	0.023942
SPP1_P647_F	0.2969127	0.025466	0.023998
FGFR2_P266_R	0.2840611	0.025501	0.023998
PLXDC2_P914_R	0.313889	0.02571	0.024151
WRN_E57_F	0.1983765	0.026518	0.024864
SMARCB1_P220_R	0.3045742	0.026584	0.024881
TCF4_P317_F	0.4015214	0.026843	0.025078
PLAU_P11_F	0.3871817	0.027113	0.025284
TRIM29_P135_F	0.2900193	0.027206	0.025325
FASTK_P598_R	0.2756202	0.027519	0.02557
SRC_E100_R	0.2959491	0.027952	0.025906
EPHA7_P205_R	0.3567689	0.027981	0.025906
CD40_P372_R	0.4159291	0.028514	0.026352
BMP2_P1201_F	0.4035216	0.02902	0.026772
MLLT4_P1400_F	0.5656896	0.029128	0.026783
DDIT3_P1313_R	0.3732504	0.029135	0.026783
MMP10_E136_R	0.3146334	0.029563	0.027127
PPAT_E170_R	0.2847052	0.029693	0.027166
APOC2_P377_F	0.3642873	0.029711	0.027166
HSPA2_P162_R	0.3957743	0.029905	0.027271
SEMA3B_P110_R	0.3268041	0.02993	0.027271
H19_P1411_R	-0.3201724	0.03002	0.027304
IL18BP_E285_F	0.3575234	0.030172	0.027394
KRAS_P651_F	0.2104301	0.030346	0.027503
CHFR_P635_R	0.3722524	0.0307	0.027775
MAD2L1_E93_F	-0.3117216	0.030937	0.027941
NBL1_E205_R	0.3125765	0.031204	0.028133
RBL2_P250_R	0.3122575	0.031259	0.028133
NGFR_E328_F	0.4148254	0.031792	0.028563
CDH17_E31_F	0.3172735	0.032266	0.028938

MAPK4_E273_R	-0.3232564	0.032516	0.029112
SRC_P297_F	0.3722464	0.032824	0.029337
EFNA1_P7_F	0.287402	0.033925	0.030268
TRIM29_E189_F	0.3114408	0.034625	0.030807
MC2R_P1025_F	-0.3282963	0.034649	0.030807
LTB4R_E64_R	0.3077343	0.034813	0.030901
SPARC_P195_F	0.3109552	0.034945	0.030964
FHIT_E19_R	0.2064062	0.035016	0.030974
MSH2_P1008_F	0.3525496	0.035525	0.031318
TNFRSF10D_P70_F	0.41493	0.035527	0.031318
GFI1_P208_R	0.3280964	0.035754	0.031465
SKI_E465_R	0.1756283	0.036254	0.03185
BCL2L2_P280_F	0.3589015	0.036443	0.031962
TUSC3_E29_R	0.4194934	0.036625	0.032067
ELK3_P514_F	0.3543913	0.036858	0.032216
RAD50_P191_F	0.3376373	0.038143	0.033283
KIT_P367_R	0.4036601	0.038299	0.033297
LIG3_P622_R	0.2841047	0.038325	0.033297
CD1A_P414_R	-0.5157086	0.038352	0.033297
HDAC1_P414_R	0.2671722	0.038713	0.033553
UGT1A1_P564_R	0.4858499	0.038802	0.033574
CPNE1_P138_F	-0.4197972	0.039415	0.034048
PI3_E107_F	-0.3589283	0.039628	0.034174
ITGB4_P517_F	0.2766015	0.040147	0.034564
CXCL9_E268_R	0.2808493	0.041398	0.035582
ABCG2_P178_R	0.2591854	0.041605	0.0357
IGFBP5_P9_R	0.3971665	0.043521	0.037282
DIO3_E230_R	0.3752744	0.043665	0.037343
EPHB3_P569_R	0.3461504	0.043968	0.03754
GABRA5_P862_R	-0.3020033	0.044296	0.037718
PTPRH_P255_F	0.32507	0.044322	0.037718
HSD17B12_P97_F	0.296843	0.044955	0.038193
GNAS_E58_F	0.3675678	0.045037	0.0382
AGXT_P180_F	-0.3389244	0.045119	0.038206

WT1_P853_F	0.4092488	0.045554	0.038512
CARD15_P302_R	0.4007519	0.046531	0.039273
AFP_P824_F	-0.3552452	0.046897	0.039518
HGF_P1293_R	-0.3649372	0.047957	0.040345
PRSS8_E134_R	0.2508256	0.048379	0.040634
MUC1_E18_R	0.2571012	0.048492	0.040662
ERCC3_P1210_R	0.5050718	0.049264	0.041243
PROM1_P44_R	0.355011	0.049789	0.041615
OSM_P34_F	0.2940318	0.050276	0.041954
FRK_P258_F	0.3015616	0.051037	0.04252
ONECUT2_P315_R	0.4406504	0.051642	0.042954
TSP50_E21_R	0.3718555	0.051777	0.042997
VAMP8_P241_F	0.2737445	0.052199	0.043212
DIRAS3_P745_F	0.3716674	0.052203	0.043212
NEU1_P745_F	-0.3497245	0.052365	0.043276
COL4A3_P545_F	0.3280449	0.053026	0.043753
ETS1_P559_R	0.4383394	0.053437	0.044022
MC2R_E455_F	-0.3912763	0.054357	0.044708
CDC25B_E83_F	0.5271728	0.054498	0.044753
OGG1_E400_F	0.3184616	0.054712	0.044857
GATA6_P726_F	0.5186343	0.054832	0.044884
DDB2_P407_F	-0.4117628	0.055252	0.045156
TUSC3_P85_R	0.3348617	0.05584	0.045565
ZIM3_P718_R	-0.3543306	0.056332	0.045894
TNFRSF1B_P167_F	0.3997545	0.057055	0.046364
MMP14_P13_F	0.2602405	0.05709	0.046364
MMP3_P16_R	-0.3215575	0.057598	0.046704
IGSF4_P454_F	0.5361385	0.057718	0.046727
CEACAM1_P44_R	0.2652868	0.057857	0.046767
HTR2A_P853_F	0.4008576	0.058333	0.047077
HLA-DPB1_P540_F	0.365383	0.058588	0.04716
PTHLH_P15_R	0.3100152	0.05863	0.04716
GPX1_E46_R	0.2993858	0.05871	0.04716
TFPI2_E141_F	0.4430774	0.058926	0.04726

GSTM1_P363_F	0.463379	0.060177	0.048188
PCDH1_E22_F	0.1230264	0.060543	0.048406
IGFBP5_E144_F	0.3078616	0.062064	0.049546
NNAT_P544_R	0.4248279	0.062614	0.049907
