# Supplementary material for: Identification of Methylated Genes Associated with Aggressive Bladder Cancer
Source: PLoS One. 2010 Aug 23;5(8):e12334. doi: 10.1371/journal.pone.0012334 (PMC2925945; doi:10.1371/journal.pone.0012334)
Supplement: Table S2 — Individual gene promoter methylation is associated with high grade in non-invasive bladder cancer. (0.05 MB PDF) [file pone.0012334.s004.pdf]

Table S2. Individual Gene Promoter Methylation is Associated with High Grade in Non-invasive Bladder Cancer

	Grade 1,2 n(%)	Grade 3 n(%)	Grade 3 OR (95% CI)	Grade 1,2 n(%)	Grade 3 n(%)	Grade 3 OR (95% CI)	Grade 1,2 n(%)	Grade 3 n(%)	Grade 3 OR (95% CI)
Total N	154	23		150	20		149	22	
**FRZB Methylation Extent**									
low (< median)	86 (92)	7 (8)	1.0 (referent)						
high (≥ median)	68 (81)	16 (19)	2.9 (1.1, 7.9)						
**KRT13 Methylation Extent**									
low (< median)				86 (95)	5 (5)	1.0 (referent)			
high (≥ median)				64 (81)	15 (19)	3.3 (1.1, 10.1)			
**HOXB2 Methylation Extent**									
low (< median)							102 (93)	8 (7)	1.0 (referent)
high (≥ median)							47 (77)	14 (23)	2.6 (0.9, 6.9)
**TP53 IHC Staining Intensity**									
Low (<3)	144 (90)	16 (10)	1.0 (referent)	140 (91)	14 (9)	1.0 (referent)	139 (90)	15 (10)	1.0 (referent)
High (3+)	10 (59)	7 (41)	7.1 (2.2, 22.5)	10 (62)	6 (38)	5.8 (1.7, 19.6)	10 (59)	7 (41)	5.1 (1.6, 16.6)

Each model is controlled for variables in columns, as well as age and gender.
