# Supplementary material for: Identification of Methylated Genes Associated with Aggressive Bladder Cancer
Source: PLoS One. 2010 Aug 23;5(8):e12334. doi: 10.1371/journal.pone.0012334 (PMC2925945; doi:10.1371/journal.pone.0012334)
Supplement: Table S3 — Primer sequences used for bisulfite pyrosequencing reactions. (0.08 MB PDF) [file pone.0012334.s005.pdf]

Table S3. Primer sequences used for bisulfite pyrosequencing reactions

Gene	Forward (5'-3')	Reverse (5'-3')	Sequencing (5'-3')	Position of region sequenced relative to transcription start site
FRZ B	AGATTATGATTTAGGTAGGATGGGGTAG	B°TCCCAAATCCTTATATCCACTTACA	TTATGATTTAGGTAGGATGG	+171 to +239
STAT5A	AGGTGTTAAGTGGGATTTATTTAAATGTG	B°TCTCCTTTCCCACTCTAAACAATAAATT	GATTTATTTAAATGTGGTAATG	-736 to -674
HOX B2	B°TTTGGGTTTTTATAATTGTATATTGTTGA	CCCTAACCACCAATTCCCTATAAT	AACCACCAATTCCCT	-125 to -92
KRT 13	GATTTTAAGGGATTTTAGGGATTATTAGAT	B°TAAAATAACTTCACCCAATTCCTACCT	AGGTATGGAAAGAGGAAG	-697 to -650

Note: B^{o} represents location of biotin label.
